# Existence and Control of Go/No-Go Decision Transition Threshold in the Striatum

**DOI:** 10.1371/journal.pcbi.1004233

**Published:** 2015-04-24

**Authors:** Jyotika Bahuguna, Ad Aertsen, Arvind Kumar

**Affiliations:** 1 Bernstein Center Freiburg and Faculty of Biology, University of Freiburg, Freiburg, Germany; 2 Computational Biology, School of Computer Science and Communication, KTH Royal Institute of Technology, Stockholm, Sweden; The Krasnow Institute for Advanced Studies, UNITED STATES

## Abstract

A typical Go/No-Go decision is suggested to be implemented in the brain via the activation of the direct or indirect pathway in the basal ganglia. Medium spiny neurons (MSNs) in the striatum, receiving input from cortex and projecting to the direct and indirect pathways express D1 and D2 type dopamine receptors, respectively. Recently, it has become clear that the two types of MSNs markedly differ in their mutual and recurrent connectivities as well as feedforward inhibition from FSIs. Therefore, to understand striatal function in action selection, it is of key importance to identify the role of the distinct connectivities within and between the two types of MSNs on the balance of their activity. Here, we used both a reduced firing rate model and numerical simulations of a spiking network model of the striatum to analyze the dynamic balance of spiking activities in D1 and D2 MSNs. We show that the asymmetric connectivity of the two types of MSNs renders the striatum into a threshold device, indicating the state of cortical input rates and correlations by the relative activity rates of D1 and D2 MSNs. Next, we describe how this striatal threshold can be effectively modulated by the activity of fast spiking interneurons, by the dopamine level, and by the activity of the GPe via pallidostriatal backprojections. We show that multiple mechanisms exist in the basal ganglia for biasing striatal output in favour of either the `Go' or the `No-Go' pathway. This new understanding of striatal network dynamics provides novel insights into the putative role of the striatum in various behavioral deficits in patients with Parkinson's disease, including increased reaction times, L-Dopa-induced dyskinesia, and deep brain stimulation-induced impulsivity.

## Author Summary

The basal ganglia (BG) play a crucial role in a variety of cognitive and motor functions. BG dysfunction leads to brain disorders such as Parkinson’s disease. At the main input stage of the BG, the striatum, two competing pathways originate. Neurons projecting on these pathways either express D1 or D2 type dopamine receptors. Because activity of D1 or D2 neurons facilitate go or no-go type decision, it is important to study the balance of the D1 and D2 neuron activity. Contrary to the common assumption thus far, recent data shows an asymmetry in striatal circuit with D1 receiving higher inhibition from D2 and fast-spiking neurons. Here, we studied the functional implications of the asymmetric connectivity between D1 and D2 neurons. Our analysis and simulations show that the asymmetric connectivity between these neurons gives rise to a decision transition threshold (DTT), as a consequence D1 (D2) neurons have higher firing rate at lower (higher) average cortical firing rates. Importantly, DTT can be modulated by input correlations, local connectivity, feedforward inhibition and dopamine. Our results suggest that abnormal changes in the DTT could be a plausible mechanism underlying the cognitive and motor deficits associated with brain diseases involving BG malfunction.

## Introduction

The basal ganglia (BG) are a set of nuclei, located at the base of the forebrain, which play a crucial role in a variety of motor and cognitive functions. The striatum is the main input stage of the basal ganglia, receiving inputs from widely distributed areas in the cortex [1], and projecting to the BG output nuclei Globus Pallidus Interna (GPi) and Substantia Nigra (SNr) via the so-called direct and indirect pathways, respectively [2]. The integration of multi-modal sensory signals with motor and/or cognitive inputs in the striatum sets the stage for action selection. Therefore, to understand the computations performed by the basal ganglia, it is of key importance to characterize the dynamical properties of striatal network activity.

Nearly 95% of the neurons in the striatum are inhibitory medium spiny neurons (MSNs). The remaining 5% are inhibitory interneurons, such as fast spiking interneurons (FSIs) and tonically active neurons (TANs) [3]. The MSNs are classified as D1 and D2 type neurons, depending on their dopamine receptor expression. Interestingly, D1 MSNs project to the GPi and SNr, forming the direct pathway, whereas D2 neurons project to Globus Pallidus Externa (GPe), forming the indirect pathway [2]. Consistent with basal ganglia anatomy and previous models [4–6], selective activation of D1 MSNs in the rat increases ambulation, whereas selective activation of D2 MSNs increases freezing behavior [7]. However, a complete shutdown of activity in either of the two neuron subpopulations might not occur in awake behaving animals [8]. In such a scenario,though, action selection could still be performed by a relative increase in the activity of one subpopulation compared to the other.

Thus far, in computational models of the interactions between direct and indirect pathways [4–6, 9], D1 and D2 MSNs have been considered as interchangeable inhibitory neuron subpopulations. In such single population models, the striatal output is controlled by the strength of cortico-striatal synaptic weights. The recurrent inhibition is not strong enough to support winner-take-all dynamics as earlier speculated [10], but may be sufficient to allow for a winner-less competition [11] and may enhance the saliency of the cortical input representation in the striatum [12]. This, however, is a highly simplistic view of the recurrent inhibition within the striatum and, as will be described below, is inconsistent with experimental data, especially given the recent findings on the recurrent connectivity in the striatum.

**Fig 1:**
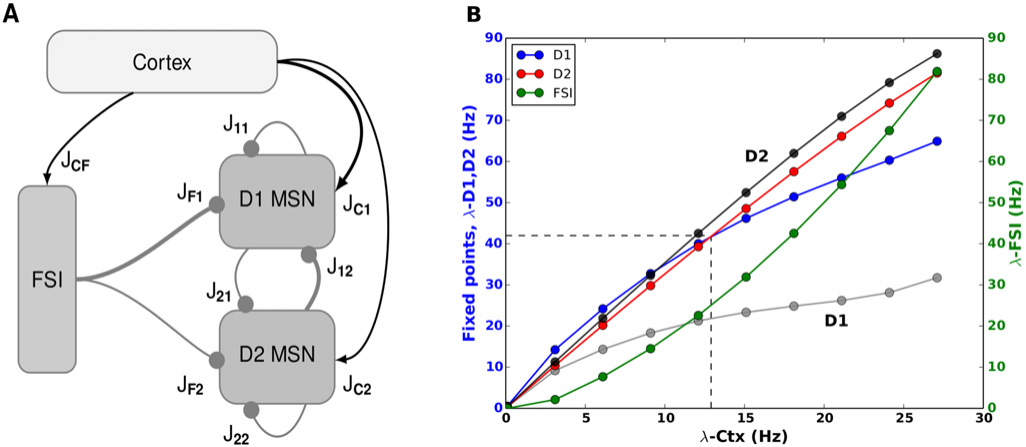
Asymmetry and decision transition threshold in the striatum. **(A)** Schematic of the striatal circuit **(B)** Steady state firing rates of the D1 (blue), D2 (red) MSNs and FSI (green) as a function of cortical inputs as estimated from the linearized mean-field dynamics model of the striatum. Here we consider the ‘multiplicative scenario’ for the extra input the the D1 MSNs. We refer to the crossover point ($\lambda_{CTX}^{tran}$), where the bias of striatal activity (Δ_*MSN*_) changes from D1 to D2 MSNs, as the decision transition threshold ≈ 13 Hz here and marked with the dashed line. The grey and black traces show the firing rates of the D1 and D2 MSNs when they received cortical inputs with the same strength, respectively. This shows that extra input to D1 MSNs is necessary to activate the ‘direct’ pathway. Otherwise, D1 MSNs cannot have higher firing rates than D2 MSNs.

Recent experiments have shown that D1 and D2 MSNs have quite different anatomical and electrophysiological properties [13]. Moreover, the striatal circuit also shows a highly specific connectivity in terms of the mutual inhibition between the MSN subpopulations [14, 15] and the feedforward inhibition from FSIs [16]. Paired neuron recordings showed that D2 MSNs make more and stronger connections to D1 MSNs, than vice versa. Furthermore, FSIs preferentially innervate D1 MSNs as compared to D2 MSNs (Fig 1A). The computational role of this specific connectivity within the striatum is not clear and cannot be inferred from previous models, which assumed a single homogeneously connected MSN population in the striatum. Specifically, it is important to identify the effect of the distinct D1 and D2 connectivities on the competition between the direct and indirect pathways.

Here we describe the effect of the heterogenous connectivity of D1 and D2 neurons on their mutual interactions using both a reduced firing rate model and numerical simulations of a spiking striatal network model. We show that the firing rates of both D1 and D2 MSNs change in a non-monotonic manner in response to cortical input rates and correlations. Interestingly, higher output rates in D1 than in D2 MSNs, and vice versa, were observed for separate, non-overlapping ranges of cortical input rate and correlation. Correlations in the input can further change the range of cortical inputs for which either D1 or D2 MSNs have the higher firing rate. Thus, we argue that the striatum acts as a threshold device for cortical input rates by changing the magnitude of the difference between firing rates of D1 and D2 MSNs, depending on the level of cortical input rates and correlations.

While the main determinant of the striatal threshold is the asymmetric connectivity among the various striatal elements, the threshold is not fixed and can be dynamically adjusted by the dopamine level, by the connectivity and firing rate of fast spiking interneurons (FSI), and by the GPe activity. That is, changes in the striatal threshold could reflect changes in the operating point of the striatum, behavioral context, and learning and reward history.

These novel insights concerning the interactions between direct and indirect pathways suggest putative mechanistic explanations for the role of striatum in cognitive deficits such as L-Dopa-induced Dyskinesia (LID), deep brain stimulation (DBS)-induced impulsivity and increased reaction times in Parkinson’s disease (PD) patients.

## Results

The striatal MSNs expressing D1 and D2 type dopamine receptors initiate the direct (‘Go’) and indirect (‘No-Go’) pathways of the basal ganglia, respectively. These two pathways converge in the GPi/SNr, their relative activity balance lets the animal choose between a ‘Go’ or a ‘No-Go’ action [4, 5]. In this framework, the decision making process in the striatum is mediated by the selective activation of one of the two MSN (D1 or D2) subpopulations. However, recent experimental data suggest that such complete shutdown of activity in either one of the two neuron subpopulations may not occur in awake behaving animals [8]. Hence, we should consider the alternative possibility that action selection is performed by a relative increase in the activity of one subpopulation compared to the other.

Therefore, to understand the effect of the recurrent, mutual connectivity and feedforward inhibition [14–16] on the relative balance of the activities in the direct and indirect pathways we studied the dynamics of the striatal network. Specifically, we evaluated the firing rates of the D1 and D2 MSNs, in response to cortical input rates and input correlations.

### D1 MSNs require overall stronger input from cortex than D2 MSNs

Experimental measurements of the mutual connectivity between striatal neurons show that D2 MSNs make more and stronger inhibitory connections on D1 MSNs than vice versa [14, 15]. In Lhx6-GFP transgenic mice, FSIs preferentially target D1 MSNs as compared to D2 MSNs [16] (however cf. [15]). This leads to the following two inequalities: J_12_ > J_21_, J_1*F*_ ≥ *J*_2*F*_, with J_12_ denoting the connection from D2 MSN to D1 MSN, J_21_ the obverse, J_1*F*_ the connection from FSI to D1 MSN and J_2*F*_ likewise to D2 MSN (cf. Table 1). These inequalities imply that if the two MSN subpopulations receive the same amount of excitatory input, D2 MSNs will always have a higher firing rate.

**Table 1:** Striatal network parameters.

Connections	Strengths
J_11_	-0.06
J_12_	-0.21
J_21_	-0.04
J_22_	-0.22
J_1*F*_	-0.09
J_2*F*_	-0.06
J_*C*1_	1.06
J_*C*2_	1.0

We confirmed this by evaluating the fixed points of the linearized dynamics of the D1 and D2 MSNs in a mean field model (Eqs 25–27, Fig 1B—grey and black traces). In our spiking network simulations this corresponded to a lower mean firing rate of the D1 population compared to the D2 population. This bias might be considered functionally useful, because for similar input strengths (i.e. when the cortex does not impose any preference for either direct or indirect pathways), the indirect pathway will dominate the striatal network dynamics and the ‘No-Go’ would be the default state of the basal ganglia.

**Fig 2:**
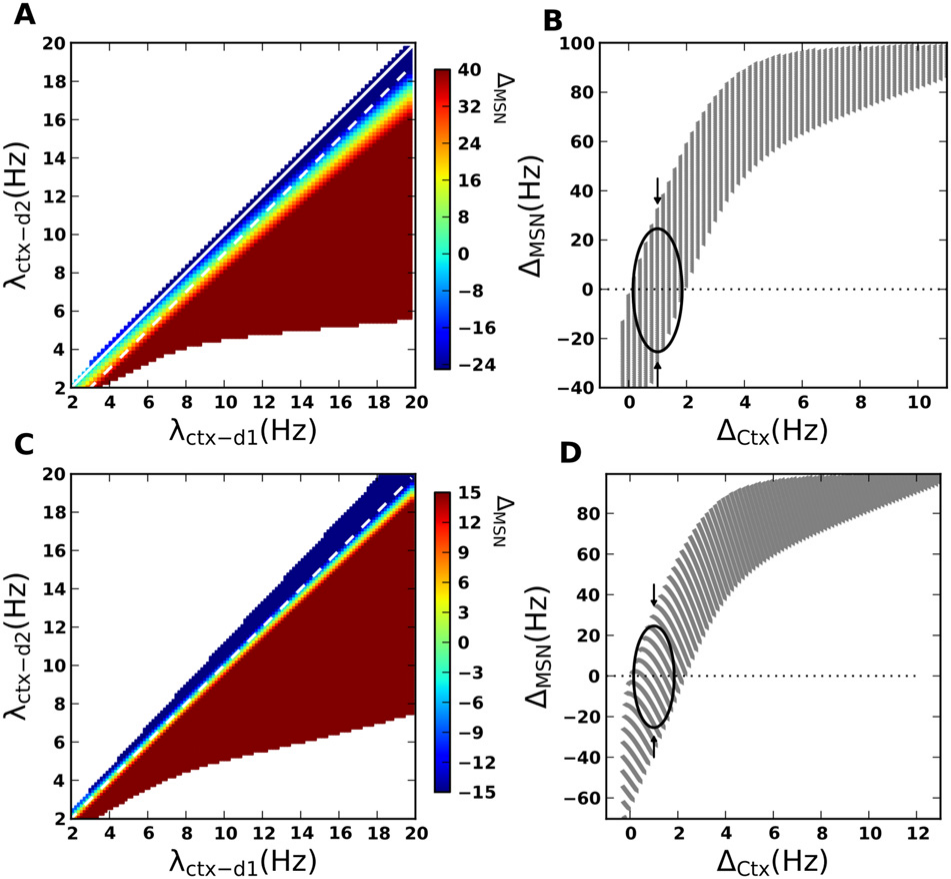
Balance of D1 and D2 MSNs firing rates as a function of cortical input rates. **(A)** Δ_*MSN*_ as a function of λ_*ctx*_*d*1_ and λ_*ctx*_*d*2_ (cf. Eq 1), scenario-I. Diagonal (marked with white line) represents the case that the two MSN populations receive equal cortical drive (λ_*ctx*_*d*1_ = λ_*ctx*_*d*2_, *X* = 0.0). With these inputs, Δ_*MSN*_ is always negative. This shows the inherent bias towards D2, due to the asymmetrical connectivity. The area below the diagonal represents the regime of higher input drive to D1 λ_*ctx*_*d*1_ > λ_*ctx*_*d*2_, *X* > 0. Off-diagonal bands represent Δ_*MSN*_ for a constant difference in cortical input rates (Δ_*CTX*_) to the two MSN populations. The dashed line marks the desirable regime of operation for the striatum, in which a systematic increase in the cortical input can reverse the sign of Δ_*MSN*_. **(B)** Δ_*MSN*_ as a function of Δ_*CTX*_ to the two MSN populations. For a constant value of Δ_*CTX*_, Δ_*MSN*_ changes depending on λ_*CTX*_ (cf. Eq 1). The oval marks the region where an increase in the cortical input rates for a constant Δ_*CTX*_ changes the sign of Δ_*MSN*_ from positive to negative, indicating a higher firing rate of the D2 MSNs. **(C)** Same as in panel **A** for the scenario-II (cf. Eq 9). In this scenario we considered *J*_*C*1_ > *J*_*C*2_, therefore, the difference in the drive to D1 and D2 MSNs scales with the difference between *J*_*C*1_ and *J*_*C*2_. Since *J*_*C*1_ > *J*_*C*2_, the diagonal itself lies in a desirable regime, where Δ_*MSN*_ changes from positive to negative for increasing cortical input rate. **(D)** Same as in panel **B** for the scenario-II.

Thus, in order for D1 MSN activity (λ_*D*1_) to exceed D2 MSN activity (λ_*D*2_), D1 MSNs must receive either stronger (*J*_*C*1_ > *J*_*C*2_), higher excitatory inputs (λ_*ctx*_*d*1_ > λ_*ctx*_*d*2_), or more excitatory synapses. Alternatively, D1 MSNs could be more excitable. Indeed, D1 MSNs have more primary dendrites and a larger dendritic arborization [13], thereby potentially receiving more inputs. Moreover, pyramidal tract neurons have stronger connections to D1 MSNs than to D2 MSNs, while intratelencephalic tract neurons innervate D1 and D2 MSNs equally. [1]. Despite this evidence, current data are not sufficient to determine the exact strength and rate of excitatory inputs to D1 and D2 MSNs. To estimate how much additional excitation would be required for D1 MSNs to have their firing rates exceed over those of D2 MSNs, we systematically varied the drive of cortical inputs to D1 and D2 MSNs and calculated the response firing rates of the two subpopulations, for the firing rate model (Fig 2). In the following we discuss two different scenarios for systematically varying the cortical excitatory drive to D1 and D2 MSNs.

#### Scenario I: Increase the relative cortical input rate to D1 MSNs

One possibility is that the cortical input rate to D1 MSNs is higher than to D2 MSNs while the strength of the cortico-striatal synapses to the two types of MSNs is the same. This results in an ‘additive’ scenario, in which we kept the strength of the cortical inputs to D1 and D2 MSNs equal (*J*_*C*1_ = *J*_*C*2_), and systematically increased the firing rate of the cortical inputs to both D1 and D2 MSNs:

$$\begin{array}{ccc} J_{C1} & = & J_{C2}\\ \lambda_{ctx\_d1} & = & \lambda_{CTX}J_{C1}+\Delta_{CTX},\;\forall \left(0\;\text{Hz}\;\leq \;\lambda_{CTX}\;\leq \;20\;\text{Hz}\right),\;\forall \left(0\;\text{Hz}\;\leq \;\Delta_{CTX}\;\leq \;13\;\text{Hz}\right)\\ \lambda_{ctx\_d2} & = & \lambda_{CTX}J_{C2},\;\forall \left(0\;\text{Hz}\;<\lambda_{CTX}<20\;\text{Hz}\right)\\ \Delta_{CTX} & = & \lambda_{ctx\_d1}-\lambda_{ctx\_d2}\\ \Delta_{MSN} & = & \lambda_{D1}-\lambda_{D2} \end{array}$$ (1)

where λ_*ctx*_*d*1_ and λ_*ctx*_*d*2_ denote the total cortical drive to the D1 and D2 MSNs, respectively and Δ_*CTX*_ denotes the extra excitatory input to the D1 MSNs. This scenario is equivalent to a state when no learning has happened and the cortex has no preference for choosing between activating D1 or D2 MSNs. Here, we considered only those parameter ranges for which neither of the two subpopulations was completely shut down by mutual and/or recurrent inhibition, because that would be a trivial solution of the network dynamics, which is also not supported by the experimental data [8].

When the two types of MSNs are driven with identical input rates (λ_*ctx*_*d*1_ = λ_*ctx*_*d*2_), the output firing rate of D2 MSNs always exceeded that of D1 MSNs, irrespective of the magnitude of the input rate (Fig 2A: main diagonal, Fig 2B: points corresponding to Δ_*CTX*_ = 0.0Hz). This shows the inherent bias of the striatal network towards higher firing rates of D2 MSNs [17], for the reasons explained above.

When the input rate to D1 MSNs exceeds that to D2 MSNs by a sufficiently large amount (here Δ_*CTX*_ ≥ 2Hz), D1 MSNs always fired at a higher rate than D2 MSNs, irrespective of the magnitude of the cortical input rate (Fig 2A: area below the dashed line, Fig 2B: area corresponding to Δ_*CTX*_ > 2.0Hz).

An interesting transitional region was observed when the input rate to D1 MSNs exceeded that to D2 MSNs by only a small amount (here: 0Hz < Δ_*CTX*_ < 2Hz). In this case, the ‘winner’ of the competition between D1 and D2 MSNs depends on the size of the cortical input rates. For small input rates to the two MSNs, D1 MSNs have the relatively higher firing rates (i.e. Δ_*MSN*_ > 0 Hz). However, as both input rates were increased, while maintaining the small difference between the two, the recurrent inhibition from D2 to D1 MSNs increased such that, beyond a certain input rate $\lambda_{CTX}^{tran}$ (here ≈ 10 Hz), it exceeded the extra small excitatory drive to the D1 MSNs, resulting in the output rate of D2 MSNs surpassing that of D1 MSNs (i.e. Δ_*MSN*_ < 0 Hz). Hence, in this transitional region with small differences in the excitatory inputs to both MSN types (0Hz < Δ_*CTX*_ < 2Hz), it was possible to have higher firing rate for either of D1 or D2 MSNs, depending on the size of the cortical input drive (Fig 2A, dashed line and 2B, set of points marked by the oval).

Thus, as a consequence of the asymmetric connectivity between D1 and D2 MSNs, the striatum can generate higher firing rates for either D1 or D2 MSNs, depending on the cortical input rates to the two, provided the difference between the two inputs is small enough. As a result, the difference between the two output rates Δ_*MSN*_ reverses as the cortical input rate is increased. In other words: due to the asymmetric connectivity between D1 and D2 MSNs, the striatum can flexibly choose between the direct and the indirect pathway, depending on the level of the cortical input firing rates. By contrast, in a regime with a large difference between the excitatory drives to D1 and D2 MSNs, the striatum would lose this ability to switch between the two, depending on the cortical input.

To understand the mechanism underlying the sign reversal we derived an expression for Δ_*MSN*_. In this input scenario the dynamics of the two MSN populations are given by:

$$\begin{array}{c} \dot{\lambda_{D1}}=-0.01\lambda_{D1}+S\left(J_{11}\lambda_{D1}+J_{12}\lambda_{D2}+J_{1F}\lambda_{FSI}+\lambda_{CTX}+\Delta_{CTX}\right) \end{array}$$ (2)

$$\begin{array}{c} \dot{\lambda_{D2}}=-0.01\lambda_{D2}+S\left(J_{21}\lambda_{D1}+J_{22}\lambda_{D2}+J_{2F}\lambda_{FSI}+\lambda_{CTX}\right) \end{array}$$ (3)

To simplify the analysis, we ignored the leak term and considered a linear transfer function, *S*(*z*) = *z* for which we calculated the fixed point rates λ_*D*1_ and λ_*D*2_. It should be noted that absolute values of parameters are used for the analysis, and to calculate λ_*D*1_ and λ_*D*2_ (Eqs 4, 5) correct sign of the various weights must be used, i.e. *J*_11_, *J*_12_, *J*_22_, *J*_21_, *J*_1*F*_, *J*_2*F*_ are negative and similarly, Δ_*CTX*_ is positive.

$$\begin{array}{c} \lambda_{D1}=\frac{\lambda_{FSI}\left(J_{2F}J_{12}-J_{1F}J_{22}\right)+\lambda_{CTX}\left(J_{22}-J_{12}\right)+J_{22}\Delta_{CTX}}{\left(J_{22}J_{11}-J_{12}J_{21}\right)} \end{array}$$ (4)

$$\begin{array}{c} \lambda_{D2}=\frac{\lambda_{FSI}\left(J_{2F}J_{11}-J_{1F}J_{21}\right)+\lambda_{CTX}\left(J_{21}-J_{11}\right)+J_{21}\Delta_{CTX}}{\left(J_{12}J_{21}-J_{22}J_{11}\right)} \end{array}$$ (5)

where Δ_*CTX*_ is again the extra excitatory input given to D1 MSNs. The response rate difference Δ_*MSN*_ = λ_*D*1_ − λ_*D*2_ now reduces to:

$$\begin{array}{c} \Delta_{MSN}=\frac{\left(J_{2F}J_{12}+J_{2F}J_{11}-J_{1F}J_{21}-J_{F1}J_{22}\right)\lambda_{FSI}+\lambda_{CTX}\left(J_{22}+J_{21}-J_{11}-J_{12}\right)+\Delta_{CTX}\left(J_{22}+J_{21}\right)}{\left(J_{22}J_{11}-J_{12}J_{21}\right)} \end{array}$$ (6)

which can be divided into two terms, *Inp*_*str*_ (Eq 7) and *Inp*_*add*_ (Eq 8):

$$\begin{array}{c} Inp_{str}=\frac{\overset{I}{\overset{︷}{\lambda_{CTX}\left(J_{22}+J_{21}-J_{11}-J_{12}\right)}}+\overset{II}{\overset{︷}{\left(J_{2F}\left(J_{12}+J_{11}\right)-J_{1F}\left(J_{21}+J_{22}\right)\right)\lambda_{FSI}}}}{\left(J_{22}J_{11}-J_{12}J_{21}\right)} \end{array}$$ (7)

$$\begin{array}{c} Inp_{add}=\frac{\Delta_{CTX}\left(J_{22}+J_{21}\right)}{\left(J_{22}J_{11}-J_{12}J_{21}\right)} \end{array}$$ (8)

Here, *Inp*_*str*_ is a measure of the effective external input, that is, cortical excitation and feedforward inhibition, each scaled by the recurrent dynamics of the striatum. The first term in *Inp*_*str*_(term I) has an effective negative contribution to Δ_*MSN*_, due to the higher effective recurrent inhibition to D1 ((*J*_11_ + *J*_12_) > (*J*_22_ + *J*_21_)). The contribution of FSI inhibition (term II) could be effectively positive or negative, depending on the ratio of feedforward (*J*_1*F*_ and *J*_2*F*_) and recurrent ((*J*_11_ + *J*_12_) and (*J*_22_ + *J*_21_)) inhibition. In our case, it is negative because (*J*_2*F*_(*J*_11_ + *J*_12_) < *J*_1*F*_(*J*_22_ + *J*_21_)). On the other hand, the additional input (*Inp*_*add*_) is a positive constant proportional to Δ_*CTX*_. That is, Δ_*MSN*_ shows a competition between *Inp*_*add*_ and *Inp*_*str*_.

**Fig 3:**
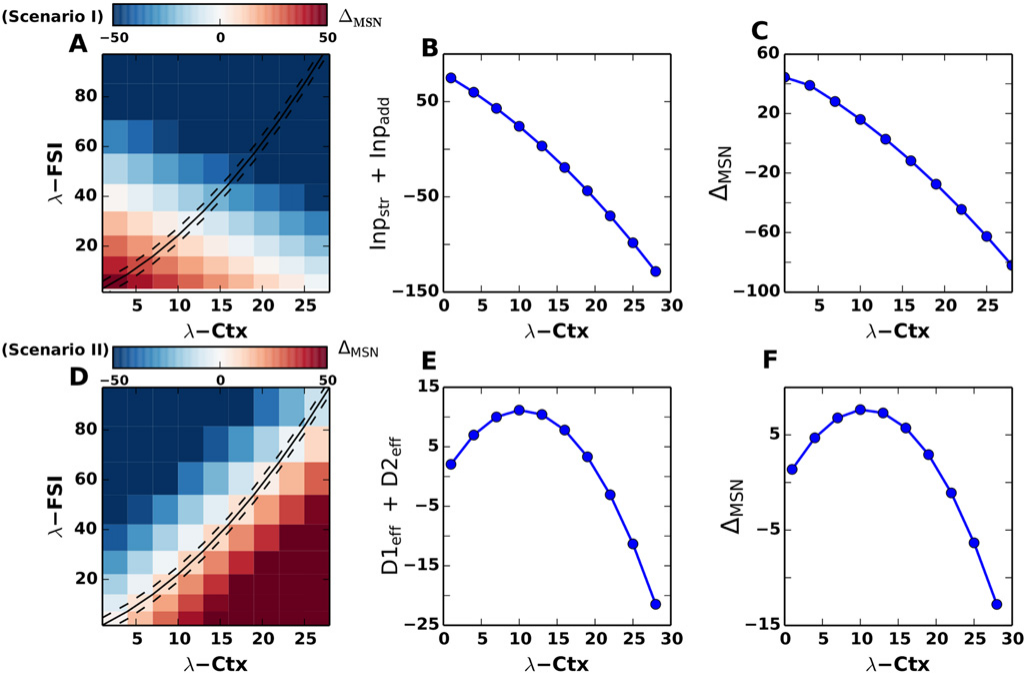
Mechanism of decision transition threshold. The mechanism of decision transition threshold is explained analytically. **(A)** Δ_*MSN*_ plotted for different values of λ_*CTX*_ and λ_*FSI*_. The dashed line refers to the case of increasing cortical excitation and feedforward FSI inhibition (as also discussed in Fig 2A). However, Δ_*MSN*_ changes from positive to negative along the rows (keeping λ_*FSI*_ constant and increasing λ_*CTX*_) and along the columns (keeping λ_*CTX*_ constant and increasing λ_*FSI*_). **(B)** Mechanism for scenario I, ‘additive’ input, Refer to Eqs 4–8. Δ_*MSN*_ = *Inp*_*str*_ + *Inp*_*add*_ plotted for increasing values of λ_*CTX*_. **(C)** Δ_*MSN*_ = λ_*D*1_ − λ_*D*2_ as calculated from Eqs 2, 3. **(D)** Δ_*MSN*_ plotted for different values of λ_*CTX*_ and λ_*FSI*_. The dashed line refers to the case of increasing cortical excitation and feedforward FSI inhibition (as also discussed in Fig 2C). However, Δ_*MSN*_ changes from positive to negative by increasing λ_*FSI*_ for a constant value of λ_*CTX*_. **(E)** Δ_*MSN*_ = *D*1_*eff*_ + *D*2_*eff*_ plotted for increasing values of λ_*CTX*_. Mechanism for scenario II, ‘multiplicative’ input. Refer to Eqs 13–14. **(F)** Δ_*MSN*_ = λ_*D*1_ − λ_*D*2_ as calculated from Eqs 21, 22.

When λ_*CTX*_ is increased, *Inp*_*str*_ increases linearly and exceeds the *Inp*_*add*_ at the value $\lambda_{CTX}^{tran}$, thereby switching the sign of Δ_*MSN*_ from positive to negative (Fig 3B). This approximative result of the linearized equation without leak was confirmed when we numerically calculated Δ_*MSN*_ from the full Eqs. 2–3 (Fig 3C).

*Inp*_*str*_ (Eq 7) is determined by both λ_*CTX*_ and λ_*FSI*_. Therefore, to understand the contribution of the activity of fast spiking neurons (assuming their overall contribution to be negative), we estimated Δ_*MSN*_ while independently varying λ_*CTX*_ and λ_*FSI*_ (Fig 3A). We found that for a fixed value of λ_*CTX*_ the sign of Δ_*MSN*_ could be reversed by an increase in λ_*FSI*_. Similarly, for a constant value of λ_*FSI*_ the sign of Δ_*MSN*_ could be reversed by an increase in λ_*CTX*_. In fact, in this scenario, Δ_*MSN*_ reverses its sign with respect to λ_*CTX*_ even in the absence of feedforward inhibition. Hence we conclude that the recurrent connectivity between the two MSN populations is the main determinant of the Δ_*MSN*_ sign reversal. Note that in Fig 3C and 3B we showed the sign reversal of Δ_*MSN*_ when both λ_*FSI*_ and λ_*CTX*_ increased in proportion (cf. black lines in Fig 3A).

#### Scenario II: Increase the relative strength of cortical projection to D1 MSNs

Instead of increasing the rate of cortical excitation, we could also increase the strength of the cortical synapses to the D1 MSNs to provide these neurons with more input. This gives rise to a second, in this case a ‘multiplicative’ scenario (Fig 2C and 2D):

$$\begin{array}{ccc} J_{C1} & > & J_{C2}\\ \lambda_{ctx\_d1} & = & \lambda_{CTX}J_{C1},\;\forall \left(0\;\text{Hz}\;<\lambda_{CTX}<20\;\text{Hz}\right)\\ \lambda_{ctx\_d2} & = & \lambda_{CTX}J_{C2},\;\forall \left(0\text{Hz}\;<\lambda_{CTX}<20\;\text{Hz}\right)\\ \Delta_{CTX} & = & \lambda_{CTX}J_{C1}-\lambda_{CTX}J_{C2} \end{array}$$ (9)

This scenario refers to a state in which cortico-striatal projection strengths have been modified in favour of D1 (and possibly also D2, but less) MSNs, due to synaptic learning. As in scenario I, we estimated Δ_*MSN*_ by systematically varying the input rate (λ_*CTX*_) and the strength of the cortico-striatal projections (*J*_*C*1_, *J*_*C*2_). Notice that in this ‘multiplicative’ scenario the difference between the input drives of D1 and D2 MSNs increases proportionally with λ_*CTX*_. Because the cortical input to D1 MSNs is now enhanced by the larger synaptic strength (*J*_*C*1_ > *J*_*C*2_), the diagonal in Fig 2C is no longer in the regime where D2 MSNs dominate (Δ_*MSN*_ < 0). Similarly to scenario I, also here also we found only a very small regime of input drive for which either D1 or D2 MSNs can have higher firing rate as cortical input rate is changed (the oval area marked in Fig 2D). Therefore, we argue that for a flexible switching between direct and indirect pathways the difference between the input rates/weights to the two MSN populations and their outputs rates should be small.

As in the ‘additive’ scenario, we performed a simplified analysis of the dynamics of Eqs 21 and 22 without leak term and a linear transfer function and found the fixed point rates λ_*D*1_ and λ_*D*2_:

$$\begin{array}{c} \lambda_{D1}=\frac{\lambda_{FSI}\left(J_{2F}J_{12}-J_{1F}J_{22}\right)+\lambda_{CTX}\left(J_{C1}J_{22}-J_{12}J_{C2}\right)}{\left(J_{22}J_{11}-J_{12}J_{21}\right)} \end{array}$$ (10)

$$\begin{array}{c} \lambda_{D2}=\frac{\lambda_{FSI}\left(J_{2F}J_{11}-J_{1F}J_{21}\right)+\lambda_{CTX}\left(J_{C1}J_{21}-J_{11}J_{C2}\right)}{\left(J_{12}J_{21}-J_{22}J_{11}\right)} \end{array}$$ (11)

Δ_*MSN*_ now reduces to

$$\begin{array}{c} \Delta_{MSN}=\frac{\lambda_{FSI}\left(J_{2F}\left(J_{12}+J_{11}\right)-J_{1F}\left(J_{22}+J_{21}\right)\right)+\lambda_{CTX}\left(J_{C1}\left(J_{22}+J_{21}\right)-J_{C2}\left(J_{12}+J_{11}\right)\right)}{\left(J_{22}J_{11}-J_{12}J_{21}\right)} \end{array}$$ (12)

This expression can be split into the effective contributions of D1 (*D*1_*eff*_) and D2 (*D*2_*eff*_), respectively:

$$\begin{array}{c} D1_{eff}=\frac{\lambda_{CTX}J_{C1}\left(J_{22}+J_{21}\right)-\lambda_{FSI}J_{1F}\left(J_{22}+J_{21}\right)}{\left(J_{22}J_{11}-J_{12}J_{21}\right)} \end{array}$$ (13)

$$\begin{array}{c} D2_{eff}=\frac{-\lambda_{CTX}J_{C2}\left(J_{12}+J_{11}\right)+\lambda_{FSI}J_{2F}\left(J_{12}+J_{11}\right)}{\left(J_{22}J_{11}-J_{12}J_{21}\right)} \end{array}$$ (14)

Cortico-striatal projections are stronger than interneuronal projections to MSNs, therefore, *J*_*C*1_ > *J*_1*F*_ and *J*_*C*2_ > *J*_2*F*_, and hence, *D*1_*eff*_ is positive and *D*2_*eff*_ is negative. At low cortical rates, ∣ *D*1_*eff*_ ∣ is larger than ∣ *D*2_*eff*_ ∣ due to the scaled cortical excitation for D1 (*J*_*C*1_ > *J*_*C*2_) and hence, the D1 type neurons dominate. However, for higher cortical driving rates, stronger feedforward inhibition to the D1 MSNs ensures that D2 MSNs exceed the firing rates of D1 MSNs, reversing the sign of Δ_*MSN*_ (Fig 3D). The values are qualitatively similar to Δ_*MSN*_ calculated numerically from the full Eqs 21 and 22 with the leak term and non-linear transfer function (Fig 3F). In general, as is evident from the Eq 12, the sign of Δ_*MSN*_ is determined by both λ_*CTX*_ and λ_*FSI*_. However, in this input scenario the relation between Δ_*MSN*_ and the firing rates λ_*CTX*_ and λ_*FSI*_ is more complex than in the ‘additive’ input scenario. Similarly to the ‘additive’ input scenario, for a fixed value of λ_*CTX*_ the sign of Δ_*MSN*_ could be reversed by an increase in λ_*FSI*_ (Fig 3D). However, for a constant value of λ_*FSI*_, an increase in λ_*CTX*_ changes the sign of Δ_*MSN*_ from negative to positive. This is because a large value of λ_*CTX*_ is needed to overcome the effect of preferential connections of the FSIs to D1 MSNs. Indeed, the higher the firing rate of FSIs, the stronger cortical input would be needed to switch the sign of Δ_*MSN*_. Note that in Fig 3F and 3E we showed the sign reversal of Δ_*MSN*_ when both λ_*FSI*_ and λ_*CTX*_ increased in proportion (cf. black lines in Fig 3D).

Although, qualitatively the two scenarios I and II yield the same result (compare Fig 2A and 2B with Fig 2C and 2D) their mechanisms are slightly different. The ‘multiplicative’ input scenario requires preferential inhibition from FSis, whereas the ‘additive’ input scenario relies on asymmetrical projections between D1 and D2 MSNs (however, see also later in subsection “Effect of symmetrical FSI projections on the DTT”). In all subsequent analyses, we used the scenario II, keeping *J*_*C*1_ > *J*_*C*2_ and maintaining identical input rate λ_*CTX*_ to both D1 and D2 MSNs. The reason is that scenario I is not suitable to compare the effect of input correlations, as this requires the input rates for D1 and D2 MSNs to be equal.

### Decision transition threshold in the striatum

**Fig 4:**
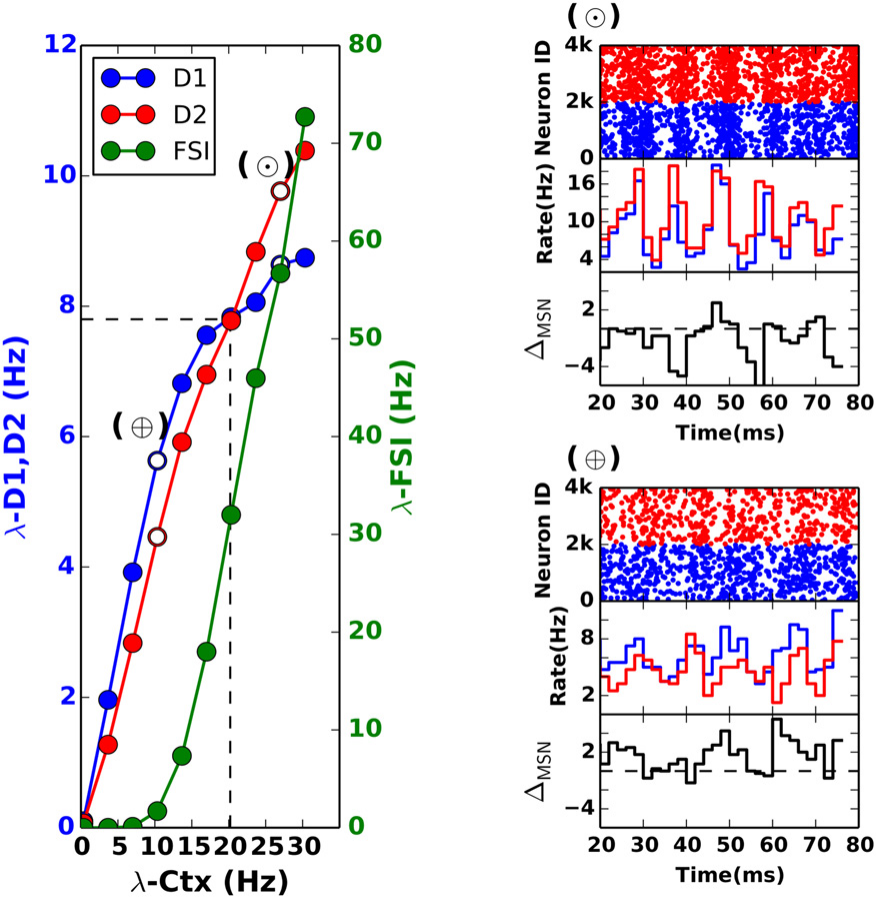
Decision transition threshold in the striatum. Mean firing rates of D1 and D2 MSNs populations for different cortical input rates with constant difference between the drives to the two populations (scenario - II cf. Eq 9). The DTT in this case is ≈ 20 Hz. The rasters and PSTH for the spiking activity for D1 and D2 MSNs are shown for two points, before and after crossing the DTT. **(A)** (top) Rasters for D1 and D2 populations at λ_*CTX*_ = 10.0Hz. (Middle) PSTHs for D1 and D2. (Bottom) Difference between the firing rates of D1 and D2 (i.e Δ_*MSN*_). Notice that the firing rate of D1 MSNs is higher than that of D2 MSNs (Δ_*MSN*_ > 0). At the decision transition threshold, the bias switches from D1 to D2.**(B)** Same as (A) but at λ_*CTX*_ ≈ 25Hz, where Δ_*MSN*_ < 0.

To obtain further insight into the activity balance of D1 and D2 MSNs, we considered a state of the striatum in which the cortico-striatal projections had been strengthened for a particular ‘Go’ task (*J*_*C*1_ > *J*_*C*2_, cf. the ‘multiplicative scenario’ for detail). This scenario is shown for the firing rate model in Fig 1B and for the spiking neural network in Fig 4). In this state, we estimated the difference between D1 and D2 MSN output rates as a function of cortical input rate. For a constant difference in synaptic strength to D1 and D2 MSNs (Δ*J* = *J*_*C*1_ − *J*_*C*2_ = const), the difference between the output firing rates of D1 and D2 MSNs (Δ_*MSN*_) changes from positive to negative as the cortical input rate increases (Figs 1B and 4). That is, the response of the striatum to a range of cortical input rates can be divided into two regimes: a regime where D1 firing rate exceeds D2 firing rate and a regime where this situation is reversed. At the transition point $\lambda_{CTX}^{tran}$, the D1 and D2 firing rates are equal. This behavior can be interpreted as a striatal bias towards the ‘Go’ pathway below a certain cortical input rate ($\lambda_{CTX}\;<\;\lambda_{CTX}^{tran}$), switching to a bias towards the ‘No-Go’ pathway for higher cortical input rates ($\lambda_{CTX}\;>\;\lambda_{CTX}^{tran}$) (cf. Figs 4A and 4B and 1B).

The cortical input rate (for a constant difference Δ_*CTX*_) at which Δ_*MSN*_ changes its sign, switching the striatum from a putative ‘Go’ to ‘No-Go’ mode, can be referred to as *decision transition threshold* (DTT). That is, the striatum acts as a threshold device, indicating the change in the cortical input rates by the sign of the difference between the firing rates of D1 and D2 MSNs—higher D1 activity reflecting lower cortical input rate and higher D2 activity reflecting higher cortical input rate. Therefore, we propose that the asymmetric striatal connectivity allows it to ‘sense’ small changes in the cortical activity reaching the striatum and to modulate the balance between ‘Go’ and ‘No-Go’ states of the basal ganglia accordingly.

Here we considered the scenario in which the sign of Δ_*MSN*_ changes from positive to negative, i.e. going from ‘Go’ to ‘NoGo’ bias. It is possible to tune the strength of FSI inputs such that initially the striatum is biased towards the ‘NoGo’ pathway and a subsequent increase in the cortical input changes the bias to the ‘Go’ pathway (cf. Fig 3A and 3D).

Previously, Lo and Wang [18] described the cortico-basal ganglia loop as a DTT for reaction time tasks. However, in their model the striatum was considered a passive unit, the threshold of which could be tuned by the strength of cortico-striatal synapses. In our model, for the first time, we describe the striatum as an active participant in deciding the level of DTT. It should however be noted, that our model explores the DTT under steady state conditions only. In this regime, the striatal network settles into stable fixed points (cf. Materials and Methods) and, therefore, the DTT is a stable network state as well. A DTT might also emerge as a transient state in the network dynamics in the presence of additional mechanisms like short term and/or long term plasticity of cortico-striatal and/or striato-striatal synapses and intrinsic plasticity of striatal neurons. Further exploration of such mechanisms is, however, beyond the scope of the present work. The presence of such dynamic DTT suggests several possible control mechanisms, that may set the bias towards either of the two pathways along the basal ganglia downstream nuclei, depending on the animal behavioral state, motivation and acquired learning. In the following sections, we describe three possible mechanisms that can modify the balance of D1 and D2 activity by modulating the level of the DTT, thereby rendering it as a dynamic variable.

In a standard feedforward model of the basal ganglia, the GPi activity can indicate the higher activity of D1 (D2) MSNs by decreasing (increasing) its activity around the baseline. However, recent data shows that the basal ganglia network is more complicated than the simple feedforward model (cf. review by [19]). In future it would be important to show whether GPi can change its activity according to the D1 and D2 MSNs firing rate differences, in a more updated model of the basal ganglia.

### Robustness of DTT

In the above, the DTT was demonstrated assuming that the firing rate of the cortical input and the difference between the input to the D1 and D2 MSNs (Δ_*CTX*_) remaines constant, but in more realistic conditions the input rate and Δ_*CTX*_ could both be random variables. Therefore, we tested the viability of the concept of DTT when the mean firing rate of the cortical inputs to the D1 and D2 MSNs was chosen from a low-pass filtered noise. In addition, the extra input to the D1 MSNs was chosen from a low-pass filtered noise. To quantify the DTT we measured Δ_*MSN*_ in pre-DTT and post-DTT regions (Supplementary S1 Fig). It is obvious that when Δ_*CTX*_ is noisy the difference between the firing rate of the D1 and D2 MSNs around the DTT decreases, however Δ_*MSN*_ in the pre-DTT and post-DTT remains large enough to be reliably measured (Supplementary S1 Fig). When the standard deviation is increased upto 50% of the mean value of the Δ_*CTX*_, both pre-DTT and post-DTT areas decreased only by 20% (Supplementary S1 Fig). Correlations in the spiking is another way of generating transient fluctuations in the input to the D1 and D2 MSNs and the effect of correlation induced fluctuations is described below (cf. subsection “Effect of cortical spiking activity correlations on the DTT”)

In addition to the input rate fluctuations, the connectivity between the D1 and D2 MSNs could also be different from what we have previously used. Therefore, we tested the robustness of the DTT for variation in the chosen values of striatal network connectivity (e.g. *J*_11_, *J*_12_ etc.). There are up to eight network connectivity parameters (cortical input to the two MSNs populations, mutual and within MSNs connectivity and MSN and FSI connectivity) which could potentially affect the existence of the DTT. We systematically varied all the parameters (except the FSI connectivity to the MSNs) by ±100% around their mean values (as used throughout this manuscript). Visualisation of this high dimensional parameter space is difficult. Therefore, here we checked if the DTT exists when only one of the parameters is varied systematically by ±100% of its mean value, while other parameters can take any value between ±100% of their mean value. A DTT is said to exist if Δ_*MSN*_ for increasing values of λ_*ctx*_ and the given parameter combination changes sign from positive to negative values exactly once. This was detected algorithmically as well as by visual inspection. Indeed, the DTT is a robust to such changes in the network connectivity and for every parameter we tested here (cortical inputs to the MSNs and within and mutual connectivity of MSNs), we can find the DTT (cf. Supplementary S2A–S2F Fig). Only for very small values of the within connectivity of the D1 and D2 MSNs (*J*_11_ and *J*_22_), we could not find a solution for the DTT. In addition, we also checked the existence of the DTT for various pairs of the network connectivity parameters (e.g. *J*_21_ vs. *J*_11_). While there are some forbidden regions in which DTT does not exist, for a large variations in the striatal connectivity, the DTT can be observed (Supplementary S2 Fig). When the existence is visualised as a scatter diagram as a function of a pair of the network connectivity parameters, the mean of these clusters lie very close to the values we have used throughout this manuscript.

### Effect of cortical spiking activity correlations on the DTT

**Fig 5:**
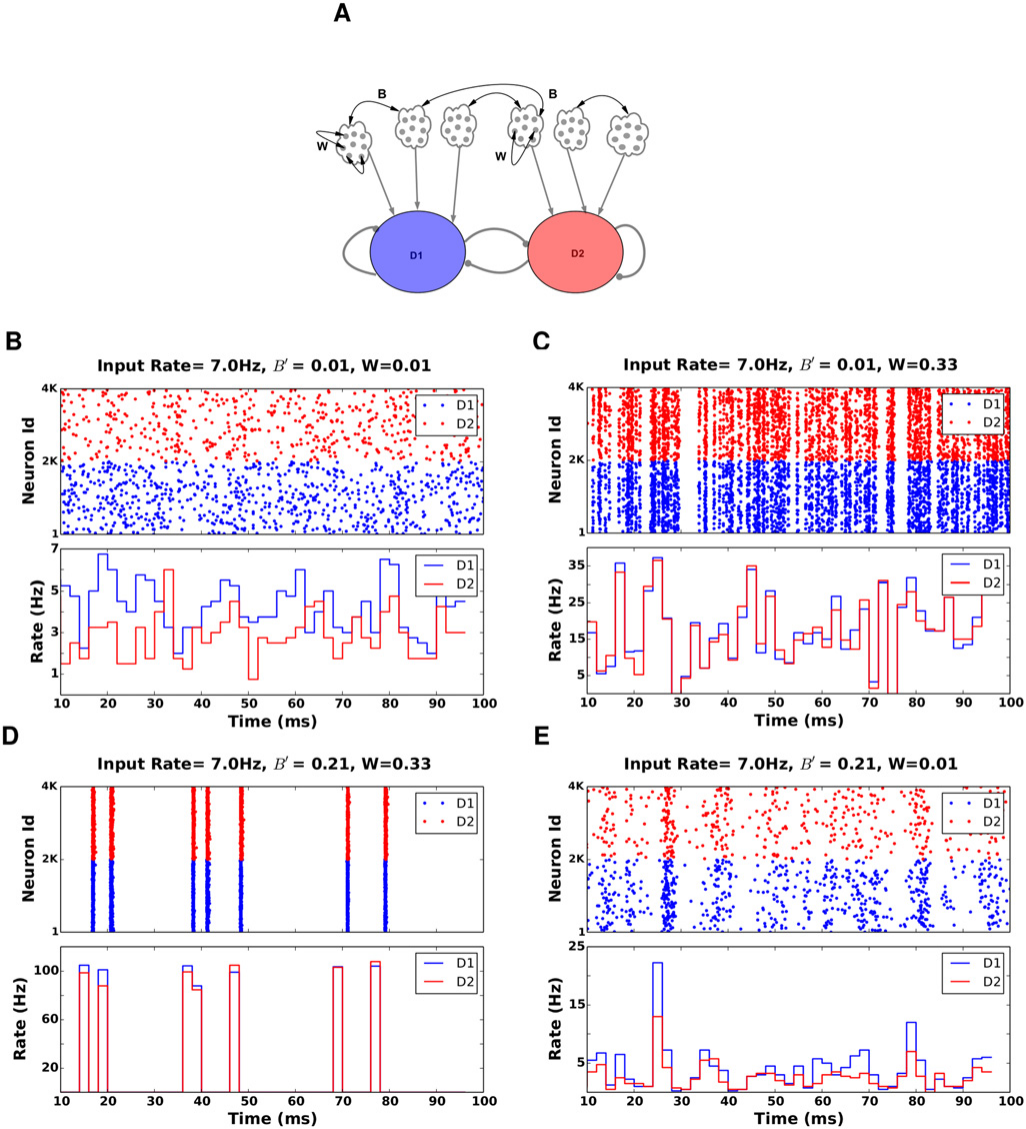
Network activity of D1 and D2 MSNs for selected values of B and W. In all examples the striatum network was configured according to the scenario-II and D1 MSNs received inputs with higher strength than D2 MSNS. Both MSN populations received cortical input at 7 Hz. **(A)** Scheme describing the two types of input correlations: *W* refers to correlations among the pre-synaptic neurons of a single MSN. *B*′ refers to correlation among the pre-synaptic neurons of two different MSNs.**(B)** *B*′ = 0.01, *W* = 0.01, raster(top) and population activity (bottom). For these inputs the activity of the D1 and D2 MSNS is uncorrelated and, therefore, exerts less effective inhibition on the other population. Because D1 MSNs are configured in the scenario-II, D1 MSNs have a higher average firing rate. **(C)** *B*′ = 0.01, *W* = 0.33. This value falls in the range of *W* < *W*_*opt*_. Here D1 operates in the ‘spike-wasting’ regime and, hence, cannot effectively inhibit D2, in spite of a higher drive from cortex. **(D)** *B*′ = 0.21, *W* = 0.33. High values of *B*′ and *W* leads to wasted inhibition due to periods of large correlated activity, followed by long periods of quiescence. **(E)** *B*′ = 0.21, *W* = 0.01. For *W* < *W*_*opt*_, B increases Δ_*MSN*_.

Task related activity in the cortex is modulated both in firing rates and in spike correlations [20]. Here we investigated how spike correlations in the cortical inputs to the striatum may affect the balance between D1 and D2 MSNs. Injection of correlated inputs to a population of MSNs requires the separation of input correlations into two categories [12]: correlations arising due to convergence of cortical projections onto individual neurons, i.e correlations within the pool of pre-synaptic neurons seen by individual MSNs (in the following referred to as ‘within’-correlations—*W*) and correlations arising due to divergence of cortical projections, i.e. correlations between the pools as seen by different MSNs, the so-called shared input (in the following referred to as ‘between’ correlations—*B*) (cf. Materials and Methods and Fig 5A). Note that because both *B* and *W* correlations arise from convergent and divergent projections of the same pre-synaptic population, between correlations are always smaller than or equal to within correlations(*B* ≤ *W* [21, 22],cf. Materials and Methods).

To understand the effect of input correlations on the balance of D1 and D2 MSN activity, we independently varied both within and between input correlations while maintaining a constant input rate (MIP model in [23]). These simulations were only performed for the spiking neural network model since modelling correlations in a mean field model is non-trivial, especially when post-synaptic neurons are recurrently connected. (cf. Materials and Methods, Fig 5A). As expected, within-correlations affected the individual firing rates in a non-monotonic manner [23] for both D1 and D2 MSNs. For weak input correlations, as D1 MSNs form stronger synapses with cortical afferents, (*J*_*C*1_ > *J*_*C*2_; scenario II), D1 MSNs spike at higher rate than D2 MSNs (Fig 5B). For high values of *W* the MSNs operated in a so called ‘spike-wasting’ regime, because the average count of coincident spikes (or the input variance) exceeded the spike threshold [23] and therefore, *W* caused no difference in the output firing rates of D1 and D2 MSNs (Fig 5C).

**Fig 6:**
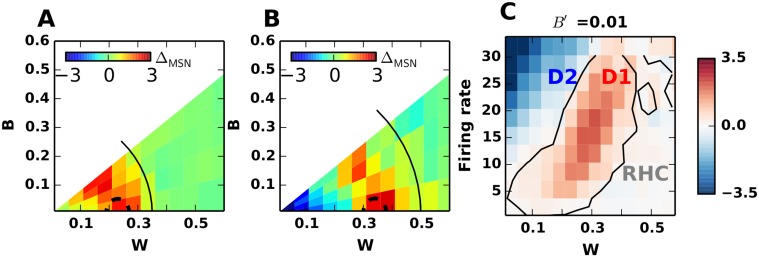
Effect of B and W input correlations on the balance of D1 and D2 activity. **(A)** Δ_*MSN*_ as a function *B* and *W* when the two MSNs population received cortical input with a firing rate of 7 Hz. Here, we considered the scenario-II and set *J*_*C*1_ = 3.6 nS, *J*_*C*2_ = 3.0 nS. At this input rate and with uncorrelated inputs (*B* = *W* = 0), D1 MSNS have higher firing rates. The black contour marks the region beyond which Δ_*MSN*_ is close to zero. All the values are concentrated below the diagonal, because of the constraint: *B* ≤ *W*. Δ_*MSN*_ varies non-monotonically as a function of *W* for a constant value of *B*. Dotted arc marks the *W*_*opt*_ for a given firing rate where Δ_*MSN*_ is maximal. For *W* ≥ *W*_*opt*_, increasing *B* decreases Δ_*MSN*_. For values *W* < *W*_*opt*_, increasing *B* increases Δ_*MSN*_. **(B)** same as in (A), for input rate of 23 Hz. *W*_*opt*_ has shifted to 0.3 as compared to 0.2 in panel **A**. **(C)** Δ_*MSN*_ as a function of *W* and input firing rate for *B*′ = 0.01. The space spanned by the input firing rates and *W* can be divided into three distinct regimes. Low *W* and high firing rates, Δ_*MSN*_ is negative(blue colours) and the output of the striatum is biased towards D2 MSNs (‘No-Go’ pathway). In the regime where *W* ≈ *W*_*opt*_, D1 MSNs have higher firing rate than D2 MSNs and the striatum output is biased towards the ‘Go’ pathway. The third regime spans across very high values of *W* in which both D1 and D2 MSNs operate in a spike wasting regime and, therefore, Δ_*MSN*_ is very low. This regime we define as ‘Region of High-Conflict’ (RHC). Because higher firing rates for D1 and D2 MSNs are observed in non-overlapping regions in the space spanned by input correlation and rates, we argue that the striatum may act as a threshold detector and signal the state of cortical inputs by raising the relative activity of D1 or D2 MSNs over the other, respectively.

That is, the difference in the firing rates of D1 and D2 MSNs (Δ_*MSN*_) changed non-monotonically as a function of *W*, reaching its maximal values for *W* = *W*_*opt*_ ≠ 0 (Fig 6A and 6B).

The effect of *W* on Δ_*MSN*_ was further modulated by the between-correlations (*B*). For *W* < *W*_*opt*_, increasing *B* resulted in a monotonic increase in Δ_*MSN*_. In this regime, *B* increased the correlation within D1 and D2 MSNs and, thereby, increased the effective inhibition of one population by the other, and vice versa. Because D1 MSNs received input with a slightly higher strength (*J*_*C*1_ > *J*_*C*2_; scenario II), *B* induced more synchrony among D1MSNs and, hence, they were better able to inhibit the D2 MSNs (Fig 5E). However, this held only over a smaller parameter regime, because by design *B* cannot exceed *W* (cf. Materials and Methods). For W ≥ *W*_*opt*_, Δ_*MSN*_ decreased monotonically with increasing B. In this regime, an increase in *B* enhanced the correlations within D1 and D2 MSNs, hence amounting to a ‘wasting of recurrent inhibition’ [12]. Increased correlations within D1 and D2 MSNs created strong but only short lasting inhibition leaving out longer lasting time windows without any recurrent inhibition (Fig 5D). In the absence of sufficiently long-lasting recurrent inhibition the difference Δ_*MSN*_ monotonically decreased (Fig 6A and 6B).

Previously, we have shown that shared inputs (*B* > 0) reduce the signal-to-noise ratio and the contrast enhancement in the striatum [12]. Our results here now show that the ‘between’-correlations could play an even more important role in initiating the action-selection process in the striatum. However, whether *B* correlations improve or impair action selection strongly depends on the ‘within’-correlations. (Fig 6). With an increase in the cortical input rate, the amount of inhibition experienced by a MSN in the striatal network also increases due to the increase in MSN firing rates. This requires more coincident excitatory inputs (i.e. higher *W*) to overcome the increased inhibition and to drive the output neurons above firing threshold. Thus, *W*_*opt*_ increases with an increase in input rates (compare Fig 6A and 6B). That is, an increase in cortical input rates broadens the dynamic range of the interaction between *B* and *W* by increasing *W*_*opt*_ (Fig 6C, also discussed in [23]).

These results show that the striatum also acts as a threshold device for cortical input correlations, in the same way we have shown above for cortical input rates. To illustrate this, we plotted Δ_*MSN*_ as a function of the cortical input rates and within correlations (*W*) for a fixed value of *B* (Fig 6C, cf. Supplementary S4 Fig). Here, we can define three regions in the space of input firing rates and input correlations for which Δ_*MSN*_ is positive, negative and zero respectively (Fig 6C), reflecting how the striatum will react to the first and second order statistics of the cortical inputs by activating the direct, the indirect or none of the two pathways. While the direct and indirect pathways reflect the striatal preference for a ‘Go’ and ‘No-Go’ action, respectively, Δ_*MSN*_ ≈ 0 could reflect a state of high conflict where it is useful to halt the decision making process until more information is available.

### Effect of Dopamine on the DTT

MSNs express D1 and D2 types dopamine receptors forming the direct and indirect pathways of the BG, respectively. Dopamine affects striatal function by modulating the intrinsic excitability of the MSNs and synaptic weights [24] and synaptic plasticity [25, 26] of the cortico-striatal projections. Because we are interested in how striatum processes cortical inputs, here we studied the effect of dopamine-induced modulation of the strength of cortico-striatal projections. Dopamine induces long term potentiation (LTP) in cortico-striatal synapses onto D1 MSNs and long term depression (LTD) in cortico-striatal synapses onto D2 MSNs [24]. This steady state effect of dopamine is often modelled by changing the weight of cortico-striatal synapses for D1 (*J*_*C*1_) and for D2 (*J*_*C*2_) [27]. Therefore, in our model we simulated the effect of dopamine depletion by decreasing *J*_*C*1_ and increasing *J*_*C*2_, whereas higher than normal dopamine was simulated by increasing *J*_*C*1_ and decreasing *J*_*C*2_.

**Fig 7:**
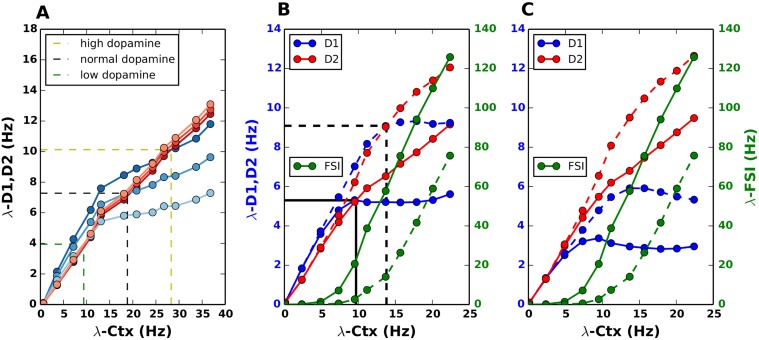
Effect of Dopamine and GPe firing rates on the balance of D1 and D2 activity. **(A)** Mean firing rates of D1 MSNs and D2 MSNs populations plotted for different levels of dopamine. Darker shades of blue (red) correspond to D1 (D2) MSN activity for higher levels of dopamine. For lower than normal levels of dopamine, the DTT shifts to the left (from ≈ 19 Hz to ≈ 9 Hz). This decreases the regime with a bias towards D1 MSNs. For higher than normal levels of dopamine, the DTT shifts to right (from ≈ 19 to ≈ 27Hz). This, in turns, increases the regime with a bias towards D1 MSNs. **(B)** Effect of GPe firing rates on the DTT. Solid lines refer to the normal state of the striatum. GPe inhibits the fast spiking interneurons, shifting the DTT to the right (from ≈ 9 to ≈ 14Hz) (dotted lines). Therefore, the regime with a D2 bias (10 < λ_*CTX*_ < 14, D2 solid red line) now has a bias towards D1 MSNs (blue dashed line) **(C)** In dopamine depleted conditions, inhibition of fast spiking interneurons via GPe is not able to switch the bias from D2 MSNs to D1 MSNs (compare dashed red and dashed blue lines).

Thus, in a low dopamine state (e.g. in PD patients), D2 MSNs received much stronger cortical input and, therefore, exerted more inhibition on to the D1 MSNs. This shifted the DTT towards lower cortical input rates (Fig 7A) reducing the regime under which the direct pathway could dominate the indirect pathway. Consistent with experimental observations and previous models [4, 5], our model suggests that dopamine depletion would (1) increase the firing rate of D2 MSNs, thereby (2) introducing a preference for ‘No-Go’ type actions.

By contrast, in a high dopamine state (e.g. as in PD patients with L-Dopa treatment) D1 MSNs received much stronger cortical input and were able to maintain higher output firing rate than D2 MSNs for a wider range of cortical inputs. That is, high dopamine shifted the DTT towards higher cortical inputs (Fig 7A) and increased the regime under which the direct pathway could dominate the indirect pathway. Consistent with experimental observations, our model suggests that an increase in steady state dopamine levels would (1) increase the firing rate of D1 MSNs [28], thereby, (2) introducing a preference for ‘Go’ type actions or dyskinesia.

In summary, therefore, synaptic effects of dopamine can be seen as a change in the DTT of the striatum.

### Effect of GPe induced disinhibition of FSI activity on the DTT

Approximately 1% of the striatal neuronal population is composed of parvalbumin expressing fast spiking interneurons (FSIs) [29]. Despite being only a small portion of the neuronal population in the striatum, they can strongly modulate MSNs activity due to their high degree of convergent and divergent connections to the MSNs. Moreover, they form synaptic connections near or at MSN’s somata as opposed to glutamergic synapses or local MSN axon collaterals, which usually connect to spines or dendritic shafts [30]. Furthermore, recent data suggests that FSIs preferentially inhibit direct pathway MSNs (D1) as compared to indirect pathway MSNs (D2) [16].

This implies that the activity of FSIs could alter the balance between the direct and indirect pathways and may even change the DTT. For instance, Fig 4A showed that for small Δ_*CTX*_, D1 MSNs fire at higher rate than D2 MSNs at low inputs, however an increase in FSI firing rate would inhibit D1 MSNs more than D2 MSNs and, hence, reduce the DTT. By contrast, a reduction in the firing rate of FSIs will have an opposite effect. Thus, FSIs could play a vital role in maintaining the default state of the striatal circuit as a ‘No-Go’ state, which could be changed to a ‘Go’ state by exciting D1 MSNs more than D2 MSNs. Alternatively, a depletion of FSIs (e.g. as seen in patients with Tourette syndrome), a reduction in their firing rate or connectivity should reverse this scenario and enforce a ‘Go’ state as the default striatum state leading to impulsivity-related behavioral symptoms.

Besides cortical inputs, other factors such as back projections from the GPe could also modulate the effect of FSIs on MSNs by modulating their firing rates (Fig 7B). The effect of GPe on FSIs was modelled in our network model as a constant amount of inhibition on FSI firing rates. An increase in GPe activity (for instance due to an increase in STN activity) would effectively release D1 and D2 MSNs from the feedforward inhibition. However, the effect will be further amplified for D1 MSNs because these neurons receive more input from FSIs than D2 MSNs do. This can shift the bias from a ‘No-Go’ to ‘Go’ state for high cortical input rate (as shown in Fig 7B, dotted lines, for λ_*CTX*_ in the range (10–15 Hz)). The same effect of FSI activity can be observed in the Fig 3D. The same effect is shown for mean field model in Supplementary S5 Fig. The temporal dynamics of such a modulation by GPe are shown in Supplementary S6 Fig for a mean field model.

Thus, an increase in the activity of the FSIs would result in shifting the DTT to higher cortical rates. We argue that increased firing rates in STN might not only maintain a tight rein on GPi to prevent a premature response in a high conflict decision, as suggested by [5], but also assist in resolving the conflict in the striatum via pallido-striatal back projections.

In the dopamine depleted condition when the DTT is very small, the effect of the GPe back projection might not be able to modulate the DTT any further (Fig 7C). Similarly, tonically active interneuron (TANs) could exploit the FSI to D1 and D2 MSNs connectivities by modulating the strength of FSI synapses onto the respective MSNs [31], thereby, changing the DTT indirectly.

### Effect of symmetrical FSI projections on the DTT

Our model is based on *in vitro* results from Taverna et al. [14] and Gittis et al. [16]. Taverna et al. [14] showed that D2 MSNs inhibit D1 MSNs more strongly in terms of numbers of projections and synaptic strengths as compared to vice versa, whereas Gittis et al. [16] showed that in the Lhx6-GFP transgenic mice, FSI has more projections to D1 than to D2, while the synaptic strengths of the projections to both MSN types are comparable. While it is unlikely that the preferential connectivity of FSIs to D1 MSNs is specific to the mouse strain (A. Gittis personal communication), another recent study by Planert et al. [15], while supporting the results of Taverna et al. [14], did not report any significant preference in the connectivity of FSIs to D1 MSNs. Thus, even though Planert et al. drew their conclusion based on only a small number of neurons, the apparent inconsistency of the data in the two studies requires a reevaluation of the connectivity of FSIs to MSNs. We, therefore, analysed the striatal dynamics for equal innervation of MSNs by FSIs.

#### Symmetrical FSI projections in the additive input scenario

In this case, the inhibition from FSIs is considered same to be the same to both MSN subpopulations (*J*_1*F*_ = *J*_2*F*_ = *J*_*F*_). Therefore, the expression for Δ_*MSN*_ in Eq 6 modifies to:

$$\begin{array}{c} \Delta_{MSN}=\frac{\left(J_{12}+J_{11}-J_{21}-J_{22}\right)\left(J_{F}\lambda_{FSI}-\lambda_{CTX}\right)+\Delta_{CTX}\left(J_{22}+J_{21}\right)}{\left(J_{22}J_{11}-J_{12}J_{21}\right)} \end{array}$$ (15)

$$\begin{array}{c} Inp_{str}=\frac{\left(J_{12}+J_{11}-J_{21}-J_{22}\right)\left(J_{F}\lambda_{FSI}-\lambda_{CTX}\right)}{\left(J_{22}J_{11}-J_{12}J_{21}\right)} \end{array}$$ (16)

$$\begin{array}{c} Inp_{add}=\frac{\Delta_{CTX}\left(J_{22}+J_{21}\right)}{\left(J_{22}J_{11}-J_{12}J_{21}\right)} \end{array}$$ (17)

**Fig 8:**
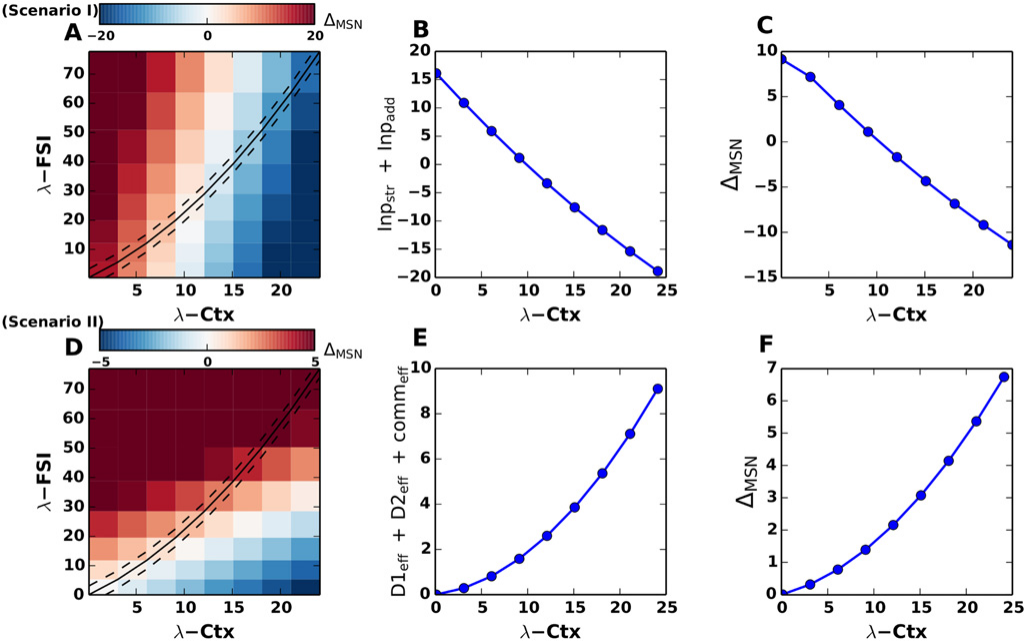
DTT in striatum with symmetrical FSI projections. **(A)** For the ‘additive’ input scenario, the striatum exhibits a DTT, in spite of the symmetrical feedforward inhibition from FSIs, refer to Eqs 15–17. A DTT is encountered not only along the diagonal (i.e increasing cortical excitation as well as feedforward inhibition from FSIs-dashed line), but also for clamped value of FSIs with increasing λ_*CTX*_. **(B)** Δ_*MSN*_ = *Inp*_*str*_ + *Inp*_*add*_ plotted for increasing values of λ_*CTX*_. **(C)** Δ_*MSN*_ = λ_*D*1_ − λ_*D*2_ as calculated from Eqs 2, 3. **(D)** In a ‘multiplicative’ input scenario, however, Δ_*MSN*_ always remains positive for symmetrical FSI projections along the diagonal. A DTT can be imposed by clamping the FSI rates at a constant value, while increasing the cortical excitation. **(E)** Δ_*MSN*_ = *D*1_*eff*_ + *D*2_*eff*_ + *comm*_*eff*_ plotted for increasing values of λ_*CTX*_. **(F)** Δ_*MSN*_ = λ_*D*1_ − λ_*D*2_ as calculated from Eqs 21, 22.

That is, when *J*_1*F*_ = *J*_2*F*_ = *J*_*F*_ the recurrent connectivity between the two MSN subpopulations (D1 and D2) becomes a more important determinant of the sign of Δ_*MSN*_. Given the nature of the MSNs inter-connectivity (*J*_12_ + *J*_11_ > *J*_21_ + *J*_22_) and the preference of FSI projections onto MSNs (*J*_*F*_ < 1), *Inp*_*str*_ remains negative and increases (decreases) with λ_*FSI*_ (λ_*CTX*_). As a consequence, for a fixed value of λ_*FSI*_, an increase in λ_*CTX*_ results in a sign reversal of Δ_*MSN*_, thereby, imposing a DTT. On the other hand, for a fixed value of λ_*CTX*_, Δ_*MSN*_ increases with increasing λ_*FSI*_. However, note that if Δ_*MSN*_ is positive, λ_*FSI*_ enhances the magnitude of Δ_*MSN*_, whereas when Δ_*MSN*_ is negative, λ_*FSI*_ attenuates the magnitude of Δ_*MSN*_ (Fig 8A). Thus, in a scenario where λ_*FSI*_ and λ_*CTX*_ are comodulated a DTT can be observed (cf. Fig 8A, solid black line and Fig 8B).

#### Symmetrical FSI projections in the multiplicative input scenario

In the multiplicative input scenario, when FSI projections to the D1 and D2 MSN subpopulations are equally strong (*J*_1*F*_ = *J*_2*F*_ = *J*_*F*_), the Eqs 13 and 14 reduce to:

$$\begin{array}{c} D1_{eff}=\frac{\lambda_{CTX}\left(J_{C1}\left(J_{22}+J_{21}\right)\right)}{\left(J_{22}J_{11}-J_{12}J_{21}\right)} \end{array}$$ (18)

$$\begin{array}{c} D2_{eff}=\frac{-\lambda_{CTX}\left(J_{C2}\left(J_{12}+J_{11}\right)\right)}{\left(J_{22}J_{11}-J_{12}J_{21}\right)} \end{array}$$ (19)

$$\begin{array}{c} comm_{eff}=\frac{J_{F}\lambda_{FSI}\left(\left(J_{12}+J_{11}\right)-\left(J_{22}+J_{21}\right)\right)}{\left(J_{22}J_{11}-J_{12}J_{21}\right)} \end{array}$$ (20)

where *comm*_*eff*_ is the common FSI feedforward inhibition to both types of MSNs. In this scenario, an increase in the λ_*CTX*_ for a constant value of λ_*FSI*_ enhances the activity of D2 MSNs and, thereby, reverses the sign of Δ_*MSN*_ from positive to negative, imposing a DTT.

By contrast, for a fixed value of λ_*CTX*_, Δ_*MSN*_ increases with increasing λ_*FSI*_ reversing the sign of Δ_*MSN*_ (Fig 8D) from negative to positive.

In a scenario where λ_*FSI*_ and λ_*CTX*_ are co-modulated, the sign reversal of Δ_*MSN*_ opposes that of the DTT (cf. Fig 8D, solid black line and Fig 8E). This is because *comm*_*eff*_ is always positive due to higher effective inhibition to D1 (*J*_12_ + *J*_11_), and hence, the sum of *D*1_*eff*_, *D*2_*eff*_ and *comm*_*eff*_ monotonically increases as shown in Fig 8D, 8E and 8F.

These results reveal an important role that FSIs can play in shaping the balance between D1 and D2 MSNs activities. In most cases, FSI activity is likely to be correlated with the cortical activity impinging onto the striatum. However, subtle changes in the correlation between the FSI and cortical activity can dramatically change striatal function. Moreover, these results also provide a possible experimental scenario to test predictions of our model. For instance, selective modulation and clamping of the activity of FSIs in a behavioural task could be used to determine whether the striatum operates in a multiplicative or additive mode, and whether FSIs have same the effective connectivity to both types of MSNs.

## Discussion

Here we studied the dynamical properties of the network of D1 and D2 type MSNs in the striatum. Specifically, we focused on the firing rates of the D1 and D2 MSNs, because the relative balance of firing in these neurons is crucial for setting the stage for action-selection in the basal ganglia and reinforcement learning. Using both mean-field analysis and spiking network simulations of D1 and D2 MSNs we show that the experimentally observed asymmetry of recurrent and mutual connections among the two types of neurons creates a ‘decision transition threshold’(DTT) in the striatum to choose between the direct (‘Go’) and indirect (‘No-Go’) pathways. Based on this DTT, the striatum performs a threshold operation on the firing rates and spike correlations reaching the striatum from the neocortex and uses the outcome to bias the action-selection.

The analysis of the striatal network dynamics revealed a narrow range of difference in cortical input rates (Δ_*CTX*_), for which it was easy to switch the balance of activity towards D1 or D2 MSNs. If Δ_*CTX*_ is negative, the striatal dynamics are stuck in a state of constant bias towards D2 MSNs. By contrast, when Δ_*CTX*_ is large and positive (more input to D1 MSNs), the striatal dynamics remain in a state of constant bias towards D1 MSNs. Thus, we predict that in order to have a flexible state in which both D1 and D2 MSNs could have higher firing rates and implement a preference towards either ‘Go’ or ‘No-Go’ actions, the D1 MSNs should receive only slightly higher cortical input than the D2 MSNs (cf. Fig 2 for details).

A more exhaustive parameter search revealed many possibilities of implementing the thresholding operation. In an ‘additive’ input scenario, a DTT emerges for a constant firing rate of FSI and increasing cortical rates and vice versa (Fig 3A) together with a third, biologically more realistic possibility of correlated firing rates of cortical inputs and FSIs.

A ‘multiplicative’ input scenario reveals a DTT for the biologically plausible case that the firing rates of the cortical inputs are correlated with the FSIs (Fig 3F and 3E). In addition, DTT can also be observed for increasing FSI firing rates and a constant cortical excitation (Fig 3D). This raises the issue of having an input-dependent DTT in the striatum, suggesting a multitude of mechanisms which could control both the existence as well as the value of the striatal DTT.

When cortical input firing rates and spike correlations are increased, while maintaining the difference in the firing rates of D1 and D2 MSNs, D1 (D2) MSNs show higher activity than D2 (D1) MSNs for low (high) input rates and spike correlations (Fig 6C). That is, in the space of input properties (firing rates and spike correlations) two regions exist for which either D1 MSNs or D2 MSNs have relatively higher output firing rates. These two regions are separated by the so called DTT. There is a third region, spanned by high input correlations (≥ 0.5) in the input parameter space, in which both neuron populations have equal firing rates. The notion of the DTT in the striatum naturally emerges as a consequence of the asymmetric connectivity between and among D1 and D2 MSNs (Figs 1 and 6). However, this does not imply that the DTT is fixed for the striatum. In fact, controlled modulation of the DTT is desired because it could provide a neuronal substrate for implementing notions like learning history, behavioral state, or motivation to perform a task. Furthermore, this view of striatal function can be extended to better understand neuronal mechanisms underlying various disorders of the basal ganglia. We identified several components of the BG network structure and activity that could potentially control and modulate the DTT.

Recent modelling work [32], shows that striatum maintains a balance of firing of D1 and D2 MSNs for different cortical input rates and this balance is maintained even when asymmetric striatal connectivity is considered. We investigated this discrepancy between our observations and those described by Damodaran et al. [32] and tuned the leaky integrate and fire neurons to closely follow the input current and output firing rate (F-I) curves of the D1 and D2 MSNs by adjusting the passive properties of the neuron (i.e. membrane capacitance, resting membrane potential and time constant) according to the values described by Gertler et al. [13](Supplementary S3A Fig). Using an asymmetric striatal network of these D1 and D2 MSNs, we show that a DTT exist for Scenario I (Supplementary S3B Fig). That is, different F-I curves and excitability of the D1 and D2 MSNs is not sufficient to abolish the DTT and create a balance of D1 and D2 output rates. Damodaran et al. [32] used a more complicated neuron model with some essential morphological features. To understand the reason for the discrepancy between our results and those described by Damodaran et al. [32], therefore, would require a more systematic analysis of the striatal connectivity and morphology and integrative properties of the MSNs, which is beyond the scope of the current manuscript.

### Modulation of the DTT by cortical input correlations

Spike correlations in cortical input to the striatum influence the DTT in a complex fashion. To better understand the effect of input correlations we separately studied the role of correlations within and correlations between the pre-synaptic pools of the MSNs. Previously, we have shown that correlations between the pre-synaptic pools of MSNs (*B*) tend to reduce the signal-to-noise ratio of the striatal response to cortical inputs [12].

On the other hand correlations within the pre-synaptic pools of individual MSNs (*W*) affect the signal-to-noise ratio of the striatal response in a non-monotonic manner. Here, we found that *W* correlations also affect the difference between D1 and D2 MSNs activity (Δ_*MSN*_) in a non-monotonic fashion (Fig 6). Interestingly, when input firing rates result in equal output firing rates of D1 and D2 MSNs, a modulation in *W* could break the symmetry and increase the firing rate of either one of the two MSN subpopulations. The value of within-correlations (*W*_*opt*_) for which Δ_*MSN*_ is maximal depends on the input firing rates and the value of between-correlations (*B*) (Fig 6).

The effect of between-correlations (or shared inputs) depends on the value of the within-correlations. In the regime *W* ≥ *W*_*opt*_, an increase in shared input (*B*) decreases the value of Δ_*MSN*_. However, in the regime *W* ≤ *W*_*opt*_, an increase in *B* increases the value of Δ_*MSN*_. Finally, for high values of input correlation ∣Δ_*MSN*_∣ is small and the two MSN populations have comparable firing rates, indicating the failure of the striatum to bias an action-selection process.

Such an operating regime could halt the decision making process in high-conflict decision-making tasks. Indeed, we predict that in high conflict choice tasks, there should be an increase of correlation in the cortical activity reaching the striatum.

### Modulation of the DTT by FSIs

The preferential connectivity of the FSIs to D1 MSNs gives the FSIs, an ability to control both the difference in the firing rate of the D1 and D2 MSNs and, hence, the DTT. For instance, an increase in FSI activity would reduce D1 MSNs activity and decrease the DTT whereas a decrease in FSI activity would tip the bias in favour of D1, and hence the ‘Go’ pathway. Such a decrease in FSI activity could be due to insufficient excitation from the cortex, or to high input from the pallido-striatal back backprojections. We suggest that selective modulation of the FSI activity, which could shift the DTT in the striatum from ‘No-Go’ to ‘Go’, could be a mechanism for decision making in a high-conflict situation. This functional concept may also help understand neuronal mechanisms underlying various behavioral phenomena. For instance, the arbitration of FSI via the STN-GPe pathway could explain longer reaction times, required to respond to a high-conflict decision. Similarly, increased impulsivity in PD patients with DBS could be explained by the fact that DBS increases GPe firing rates [33], which would decrease the activity of FSIs by pallido-striatal backprojections, resulting in lowering the DTT.

Being the primary source of feedforward inhibition, FSIs play an important role in both the existence and the actual value of the DTT. For example, we find that a preferential inhibition of D1 MSNs is required for a DTT to be present for ‘multiplicative’ input. By contrast, an ‘additive’ scenario reveals a DTT, irrespective of the asymmetry of FSI projections onto MSNs. We showed that even in the ‘multiplicative’ input mode the striatum can exhibit a DTT, by clamping the FSIs rates, while increasing cortical input rates. We conclude that FSI activity plays a prominent role in determining the DTT in the striatum, underlining once more the functional importance of pallido-striatal backprojections to FSIs.

### Modulation of the DTT by dopamine

At the synaptic level dopamine has been described to increase (decrease) the strength of cortico-striatal synapses onto D1 (D2) MSNs. This differential effect of dopamine provides a powerful mechanism to control the DTT and difference between the activities of the two neuronal subpopulations activities and, hence, the DTT. For instance, a dopamine induced increase in synaptic inputs to D1 MSNs shifts the DTT in the favour of D1 MSNs, and thereby increasing the cortical input regime for a ‘Go’ bias. This, in fact, could be a putative mechanism by which external administration of L-Dopa induces dyskinesia as a prominent side-effect. By contrast, a reduction in dopamine level reduces the regime for a ‘Go’ bias, while increasing the regime for a ‘No-Go’ bias. Thus, low dopamine results in higher firing rates in D2 MSNs, possibly manifesting in akinetic symptoms and inducing oscillations in the GPe-STN network [34].

Recent experimental data suggest that the activation of ‘Go’ and ‘No-Go’ pathways might not be exclusive *in vivo* awake behaving animals. At least at the population level, both D1 and D2 MSNs have been shown to be co-activated and neither of the two pathways were completely shutdown [8]. Consistent with these experimental findings, both D1 and D2 MSNs in our model are co-activated, however, the two populations differed in their firing rates. We note that here our goal is not to reproduce the experimental results of Cui et al. 2013 [8] which involved behaving mice performing self-paced lever pressing to retrieve the reward. Apart from the obvious simplicity of our network model, we think there might be an additional reason for this. In our model, we considered the striatum as unit representing a single action. Indeed, Cui et al. 2013 [8] suggest that a non-selective global deactivation of D2 MSNs in the striatum would abolish suppression of most unwanted motor programs and, hence, lead to hyperkinesia. It can be argued that our model represents such a global activation and deactivation of D1 and D2 in the striatum. The concept of a DTT, if extended to include multiple action channels, could be used to more closely address the results reported in [8].

### Implications of asymmetry in the striatal circuit

**Fig 9:**
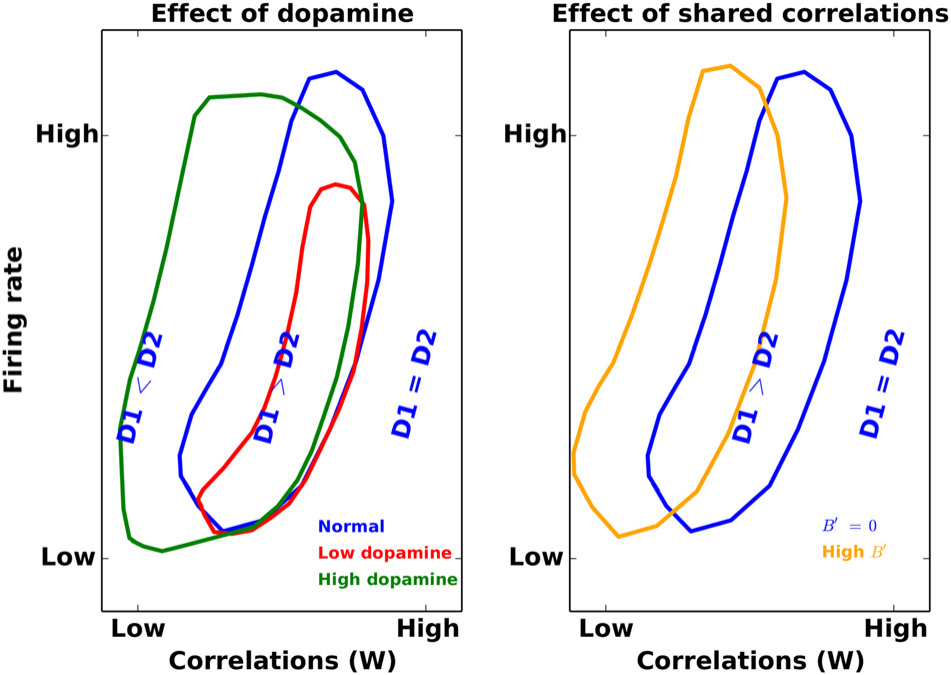
Modulation of the DTT in the striatum. (**Left**) Each contour encloses an area in the parameter space of input correlation (W) and input firing rates, in which the firing rate of D1 MSNs exceeds that of D2 MSNs. The blue contour represents the healthy state of the striatum. Loss of dopamine results in a decrease in the strength of excitatory inputs to the D1 MSNs and, therefore, the blue contour shrank to the red contour indicating that for a large range of firing rates and correlations D2 MSNs have higher firing rate than D1 MSNs. The green contour on the other hand depicts a state in which there is a high level of dopamine in the striatum (e.g. in PD patients on L-Dopa treatment) because excessive dopamine increases the overall excitability of D1 MSNs, thereby expanding the region in which D1 MSNs have a higher firing rate. We refer to the region in which the firing rates of D1 and D2 MSNs are comparable as a region of high-conflict. (**Right**) The blue contour is the same as in the left panel. The orange contour shows how the increase in shared correlation (*B*′) could increase the region of high-conflict (RHC).

Thus far, the striatum has been considered as a homogenous structure, with a weak and homogeneous inter-connectivity. Anatomical evidence, however, suggests that there is an inherent asymmetry in the striatal circuit in terms of mutual connections between D1 and D2 MSNs, as well as in feedforward projections from FSIs. In Fig 2, we showed that this asymmetry, for an appropriate activity regime, has the ability to reinforce the cortical bias (bias towards D1 at low cortical rates) or override it (bias towards D2 at high cortical rates). Taking cortical input correlations into consideration, these dynamics become even more complex, as is summarized in Fig 9. Low dopamine levels decrease the regime with D1 bias, while increasing the regime with D2 bias. Alternatively, an increase in dopamine levels increase the area for a D1 bias, while decreasing the area for a D2 bias. An increase in between-correlations in cortical input (*B*), however, pushes the contour towards the top left corner in Fig 9 (towards higher firing rates and lower values of within-correlation (*W*)), thereby enlarging the area of conflict (RHC). We therefore suggest, that the asymmetry in the striatal circuit is useful to cover different regimes of D1 and D2 bias, accommodating characteristic features of cortical input, including rates, input correlations and local dopamine levels.

Given the *in vitro* experimental conditions [14, 15], the measurement of the connectivities within and between D1 and D2 MSNs could be biased. In more realistic *in vivo* conditions, the connectivities of D1 and D2 MSNs may turn out to be different. In particular, if D1 and D2 MSNs would not differ in their connectivities and membrane properties, there is no reason to consider the striatum as a two population network and the role of action selection would need to be performed in the downstream nuclei. Such a single population striatum could initiate this process by ‘winner-less competition’ [11] or by enhancing the contrast of the input [12]. If the D1 and D2 MSNs would only differ in their membrane properties, then our analysis of asymmetric connectivity between the two neuron subpopulations would still be valid, because the effect of different neuron transfer functions could be reduced to a difference in effective connection strengths. In the extreme case, when D2 MSNs would receive more inhibition than D1 MSNs, our analysis remains valid, but we would have to reverse the interpretation.

### Implications for the understanding brain disorders involving the basal ganglia

Many disorders in the basal ganglia are related to the imbalance of its functional pathways. PD is thought to be a manifestation of dopamine depletion in the form of a domination of indirect and hyperdirect pathways, while weakening the direct pathway. Dyskinesia, on the other hand, is correlated with hyperactivity of the direct pathway and reduced activity of the indirect pathway [35]. The Tourette syndrome is correlated with decreased activity of FSIs [36]. This (dis)balance of striatal activity can be systematically modeled using the concept of the DTT. In fact, our model provides multiple neuronal mechanisms and a mechanistic understanding of several brain disorders by linking their specific pathophysiology to the DTT. Specifically, we explored three factors that may influence the DTT.

In summary our results show that the striatum, with its asymmetric connectivity among and between D1 and D2 MSNs, can act as a threshold device, indicating the increase in cortical input firing rates and correlations by increasing the relative firing rates of D1 and D2 MSNs. The DTT (basically, a threshold on the difference between the two) is a dynamic variable which may represent behavioral state, learning history and motivation level of the animal. Various mechanisms e.g. feedforward inhibition, dopamine, GPe backprojections, tonically active neurons exist that can modulate the DTT and thereby, provide the striatum with a rich computational repertoire.

We arrived at this functional description of the striatum as a DTT by considering the striatal network dynamics emerging from including low-level properties such as synaptic weights and connection probabilities. Moreover, we included several factors that can modulate the DTT level by affecting chemical imbalances and changes in neuronal properties. Taken together, we provide a high-level functional model of the striatum, which can be easily linked to low-level properties. Typically, the striatum is included in large-scale functional models [5], acting according to winner-take-all dynamics; an idea no longer supported by experiments [10]. Instead, we argue that our functional description of the striatum provides a more biologically realistic basis for large-scale functional models of basal ganglia function.

### Model predictions and explanation of experimental data

#### High-conflict decision making

The cognitive concept of conflict is described as a quantity that should increase with the absolute amount of activation and the number of competing representations [37]. In this condition, it is essential for the action selection system to stall the decision for further deliberation. A stalled decision in our model implies that the activity in the direct and indirect pathways are comparable i.e. Δ_*MSN*_ ≈ 0 (in our model Δ_*MSN*_ ≈ 0 for high cortical firing rates and correlations (Fig 6A)). Especially an increase in *B* expands the region in which Δ_*MSN*_ ≈ 0 (Fig 9). Therefore, we predict that high-conflict tasks should be associated with an increase in correlations in the activity of cortico-striatal projection neurons. Recent experimental data shows higher cortical firing rates in high-conflict situations [38]. According to our model this is not sufficient for decision stalling, because it would lead to a higher firing rate in D2 MSNs. Moreover, increased cortical rates should be accompanied with an increase in correlations.

After stalling the decision, at some point a decision needs to be made in favor of either the direct or the indirect pathway. A rather simple way to resolve the conflict would be to change the cortical firing rates and correlations (cf. Fig 9). The pallido-striatal backprojections may also provide alternative mechanisms to resolve conflict and facilitate the decision making.

#### L-Dopa induced Dyskinesia

In our model, an increase in the steady state level of dopamine changes the DTT to higher cortical input rates, thereby increasing the region in which D1 MSNs can have their firing rate exceed those of D2 MSNs (Fig 7A). This observation could be used to explain the dyskinesia often reported in patients with PD on L-Dopa therapy (L-Dopa Induced Dyskinesia—LID). Undesired involuntary movements characteristic of LID can be attributed to the frequent undesired activation of D1 MSNs (direct pathway). This could be a direct consequence of a shift in DTT towards ‘Go’, which would result in a decrease in GPi activity during LID. Indeed, injection of dopamine agonists in MPTP treated nonhuman primates induced dyskinesia and showed a marked decrease in GPi firing rates [35].

#### Increased reaction time in PD patients

According to our model, in a low dopamine state, there is a very small parameter regime in which D1 MSNs can have higher firing rate than D2 MSNs (Fig 7A). Therefore, we propose that in a low dopamine state even low-conflict tasks might require the arbitration by pallido-striatal backprojections via FSIs (as shown in Fig 7B and S5B), thereby increasing the average reaction time for voluntary actions in Parkinson’s disease patients.

#### Akinesia in low-dopamine state

In our model, a decrease in the steady state dopamine level results in an increase in the range of cortical input for which D2 MSNs have a higher firing rate than D1 MSNs. This might give rise to induced akinesia observed in dopamine-depleted conditions. Such a bias towards ‘No-Go’ may have a two-fold effect on the basal ganglia output. First, it would introduce an insufficient ‘Go’ bias in the GPi (firing rates do not decrease sufficiently) and second, in extreme dopamine depleted conditions it might introduce a strong inhibition on GPe, which can instigate beta band oscillations in the GPe-STN loop [34]. Furthermore, unlike in normal dopamine conditions, the STN-GPe loop is also insufficient to shift the balance of activity towards D1 MSNs (Figs 7C and S5C).

#### Selective inhibition of FSIs causes dystonia in animal models

In several brain disorders, the number of FSIs and/or their connectivity to MSNs are altered. For instance, FSI count is reduced in Tourette syndrome and dystonia [39, 40]. Because FSIs preferentially connect to D1 MSNs, according to our model the loss of FSIs or their connectivity to MSNs would increase the activity in the direct pathway, compared to the indirect pathway (Fig 7B and 7C) and shift the DTT to higher values. This might underlie the symptoms of Tourette’s syndrome and dystonia. This explanation is partially supported by recent experimental findings which show that the symptoms of dystonia could be evoked in an animal model by a selective decrease of the firing rate of FSIs [36].

#### Re-wiring of FSIs towards D2 MSNs in PD animal models may be a compensatory mechanism

In our model, based on the experimental data, FSIs preferentially innervate D1 MSNs [16] in order to maintain the default state of bias as ‘No-Go’ under equal cortical drive, while allowing the cortical activity to switch the balance of D1 and D2 MSNs activity. Interestingly, in the mouse model of PD, three days after dopamine depletion, the FSIs doubled their connectivity to D2 MSNs as compared to D1 MSNs [41]. Our model suggests that dopamine depletion reduces the DTT such that the striatum mostly operates in a ‘No-Go’ state and, hence, cortical activity no longer suffices to switch the balance of D1 and D2 MSNs activity. In our model, an increase in the FSI to D2 MSNs connectivity will shift the DTT back in favour of D1 (Fig 4B). Therefore, we argue that the experimentally observed rewiring of the FSIs to D2 MSNs may be a compensatory mechanism to maintain striatal function in a low dopamine state.

#### Increased impulsivity in PD patients with deep brain stimulation

Impulsivity could be considered as a too frequent activation of D1 MSNs or equivalently, a reduced DTT of the striatum. Deep brain stimulation (DBS) in the STN is known to increase GPe firing rates [33]. Such increased GPe firing rates would impose a sustained inhibition of FSIs, thereby shifting the DTT to higher cortical input rates and expanding the regime with a bias towards the ‘Go’ pathway, which may manifest itself as impulsivity at the behavioral level. Notice,that a similar trend was discussed in LID in Fig 7A. Therefore, we predict that striatal dynamics should show similar characteristics during impulsivity and LID. Indeed, there is evidence for the potential mechanistic overlap between behavioral ICDs (impulse control disorders) and motor (dyskinesia) dopaminergic ramifications (cf. review in [42]).

## Materials and Methods

The schematic of the striatal circuit is shown in Fig 1. The parameters considered for the rate model are included in Tables 1 and 2.

**Table 2:** Parameters for Eq (24).

Parameter	Description	Value
*N*_1,2_	Number of neurons in the population	2000
*N*_*fsi*_	Number of neurons in the population	80
*ρ*_11,21,22,12_	% Connectivity	0.26,0.07,0.36,0.27 [14]
*ρ*_*d*1*fsi*,*d*2*fsi*_	% Connectivity	0.54,0.36 [16]
*IPSC*_11,21,22,12_	% Connection strength	42pA,107pA,107pA,133pA [14]
*R*_*msn*_	Input resistance	238MΩ [14]
*τ*_*msn*,*fsi*_	Synaptic time constant	12msec,8msec [14, 16]

### Mean field model

The mean population dynamics of the striatal circuit for scenario II were modelled using coupled non-linear differential equations:

$$\begin{array}{c} \dot{\lambda_{D1}}=-0.01\lambda_{D1}+S\left(J_{11}\lambda_{D1}+J_{12}\lambda_{D2}+J_{1F}\lambda_{FSI}+J_{C1}\lambda_{CTX}\right) \end{array}$$ (21)

$$\begin{array}{c} \dot{\lambda_{D2}}=-0.01\lambda_{D2}+S\left(J_{21}\lambda_{D1}+J_{22}\lambda_{D2}+J_{2F}\lambda_{FSI}+J_{C2}\lambda_{CTX}\right) \end{array}$$ (22)

The values of parameters used in Eqs 21 and 22 are listed in the Table 1.

The non-linear transfer function of the neuron *S*(*z*) has a sigmoidal function of the form:

$$\begin{array}{c} S\left(z\right)=\frac{z}{\sqrt{z^{2}+1}} \end{array}$$ (23)

The parameters J_11_, J_12_, J_21_, J_22_ are calculated by considering the effective input received by a neuron in the population. This can be quantified as:

$$\begin{array}{c} J_{xy}=N_{y}\times \rho_{xy}\times IPSC_{xy}\times R_{x}\times \tau_{xy} \end{array}$$ (24)

with *x* and *y* either 1 or 2. These parameters are listed in Table 2.

This measure calculates the relative inhibitory strength of projections between D1 and D2: although the self connectivity of D1 is comparable to the number of projections it receives from D2 (*ρ*11 = 0.26, *ρ*_12_ = 0.27), since D2 makes stronger inhibitory connections than D1, J_12_ is larger than J_11_.

#### Stability of the striatal circuit

Linearizing around the fixed point with the Jacobian matrix:

$$\begin{array}{c} \left[\begin{array}{cc} \left(\frac{1}{\sqrt{z_{1}^{2}+1}}+\frac{2z_{1}^{2}}{{\left(z_{1}^{2}+1\right)}^{1.5}}-0.01\right)*J_{11} & \left(\frac{1}{\sqrt{z_{1}^{2}+1}}+\frac{2z_{1}^{2}}{{\left(z_{1}^{2}+1\right)}^{1.5}}\right)*J_{12}\\ \left(\frac{1}{\sqrt{z_{2}^{2}+1}}+\frac{2z_{2}^{2}}{{\left(z_{2}^{2}+1\right)}^{1.5}}\right)*J_{21} & \left(\frac{1}{\sqrt{z_{2}^{2}+1}}+\frac{2z_{2}^{2}}{{\left(z_{2}^{2}+1\right)}^{1.5}}-0.01\right)*J_{22} \end{array}\right] \end{array}$$ (25)

where

$$\begin{array}{c} z_{1}=J_{11}\lambda_{D1}+J_{12}\lambda_{D2}+J_{1F}\lambda_{FSI}+J_{C1}\lambda_{CTX} \end{array}$$ (26)

$$\begin{array}{c} z_{2}=J_{21}\lambda_{D1}+J_{22}\lambda_{D2}+J_{2F}\lambda_{FSI}+J_{C2}\lambda_{CTX} \end{array}$$ (27)

allows to analyze the stability by calculating the eigenvalues of the fixed point. We found the eigenvalues to be real and negative for all values of the fixed points.

### Network Simulations

The striatal network model was based on the spiking network model of the striatum as described in [12], except that the network connectivity was not considered to be homogeneous as in [12]. The simulations were carried out in NEST [43] with networks of 4000 MSNs (2000 D1, 2000 D2) and 80 Fast Spiking Interneurons (FSI), consistent with the estimated ratio of MSN and FSI neurons [44, 45]. The neuron model parameters for FSIs and MSNs are listed in Table 3.

**Table 3:** Parameters for model neurons in network simulations.

Parameter	MSN(D1,D2)	FSI
Number of neurons	4000	80
*V*_*rest*_(mV)	-80 [45]	-80 [45]
*V*_*exc*_(mV)	0	0
*V*_*inh*_(mV)	-64 [48]	-76 [49]
*V*_*th*_(mV)	-45 [50]	-54 [49]
*τ*_*exc*_(ms)	0.3	0.3
*τ*_*inh*_(ms)	2	2
*C*(pF)	200 [13]	500
*G*_*rest*_(nS)	12.5 [51]	25

D1 MSNs, D2 MSNs and FSIs received independent excitatory Poisson inputs, mimicking the background cortico-striatal inputs. The cortical input parameters are summarized in Table 4. Connection probabilities for D1 MSNs, D2 MSNs and FSIs were taken from [14, 16], also listed in Table 5.

**Table 4:** Cortical input to striatal neurons.

Target	Background input rate (Hz)	Peak conductance(nS)
D1	2500	3.6
D2	2500	3.0
FSI	2500	5.0

**Table 5:** Inhibitory input to striatal neurons.

Source	Target	Probability	Peak conductance (nS)	Delay(ms)
D1	D1	0.26 [14]	0.5	2
D1	D2	0.07 [14]	1.0	2
D2	D2	0.36 [14]	1.0	2
D2	D1	0.27 [14]	1.2	2
FSI	D1	0.54 [16]	2.5 [50]	1
FSI	D2	0.36 [16]	2.5 [50]	1

The leaky-integrate-and-fire (LIF) neuron model was used to simulate the neurons in the network with the subthreshold dynamics of the membrane potential V^*x*^(t) described by:

$$\begin{array}{c} C^{x}{\dot{V}}^{x}\left(t\right)+G^{x}\left[V^{x}\left(t\right)-V_{rest}^{x}\right]=I^{syn}\left(t\right) \end{array}$$ (28)

$$\begin{array}{c} I^{syn}\left(t\right)=I^{D1}\left(t\right)+I^{D2}\left(t\right)+I^{FSI}\left(t\right)+I^{Ctx}\left(t\right) \end{array}$$ (29)

where *x* ∈ D1, D2, FSI. In the above equations, *I* ^*syn*^(*t*) describes the total cortical excitatory, recurrent and feedforward inhibition to a neuron, *C* ^*x*^ is the membrane capacitance, *G* ^*x*^ is the leak conductance, and *V*_*rest*_ is the resting membrane potential. When the membrane potential of the neuron reached *V*_*th*_, a spike was elicited and the membrane potential was reset to *V*_*rest*_ for a refractory duration (*t*_*ref*_ = 2 ms.)

An alpha function was used to model the excitatory synaptic input received by D1, D2 and FSI:

$$\begin{array}{c} g_{exc}^{x}\left(t\right)=\{\begin{array}{c} J_{exc}^{x}\frac{t}{\tau_{exc}}e^{1-\frac{t}{\tau_{exc}}}\;for\;t\geq 0\\ 0\;for\;t<0 \end{array} \end{array}$$ (30)

where *x* ∈ D1, D2, FSI. The rise times for the excitatory inputs *τ*_*exc*_ was set to be identical for all neurons. In addition to excitatory input from the cortex, D1 and D2 MSNs received (self and mutual) recurrent inhibition as well as feedforward inhibition from the FSIs. The corresponding inhibitory conductance changes were modelled as:

$$\begin{array}{c} g_{inh}^{x}\left(t\right)=\{\begin{array}{c} J_{inh}^{x}\frac{t}{\tau_{inh}}e^{1-\frac{t}{\tau_{inh}}}\;for\;t\geq 0\\ 0\;for\;t<0 \end{array} \end{array}$$ (31)

where *x* ∈ D1, D2. The rise times *τ*_*inh*_ for all inhibitory synaptic conductance transients were set to have identical values. Assuming the synaptic strengths as fixed, the total excitatory conductance $G_{exc,i}^{x}(t)$ in a MSN *i* was given by

$$\begin{array}{c} G_{exc,i}^{x}=\sum_{m\in K_{i}^{x}}\sum_{n}g_{exc}^{x}\left(t-t_{mn}^{Ctx}\right) \end{array}$$ (32)

where *x* ∈ D1, D2. The sequence of spikes (*n*’*s*) impinging on a particular-synapse *m* are added by the inner sum, while the outer sum runs over all such excitatory synapses *m* in the set $K_{i}^{x}$ projecting onto neuron *i*. The set $t_{mn}^{Ctx}$ represents the spike times of the excitatory neuron *m*.

The total inhibitory conductance $G_{inh,i}^{x}(t)$ in a D1 or D2 MSN *i* was given by

$$\begin{array}{ccc} G_{inh,i}^{x} & = & \sum_{m\in K_{i}^{FSI}}\sum_{n}g_{inh}^{FSI}\left(t-t_{mn}^{FSI}-\Delta^{FSI}\right)+\sum_{m\in K_{i}^{D1}}\sum_{n}g_{inh}^{D1}\left(t-t_{mn}^{D1}-\Delta^{D1}\right)\\ & & +\;\sum_{m\in K_{i}^{D2}}\sum_{n}g_{inh}^{D2}\left(t-t_{mn}^{D2}-\Delta^{D2}\right) \end{array}$$ (33)

where *x* ∈ D1, D2 and $K_{i}^{FSI}$, $K_{i}^{D1}$, $K_{i}^{D2}$ refer to the pre-synaptic FSIs, D1 and D2 projecting onto neuron *i*, respectively. The transmission delays of Δ^*FSI*^ and Δ^*D*1^, Δ^*D*2^ were fixed to 1ms and 2ms, respectively. The total synaptic current to a MSN *i* was

$$\begin{array}{c} I_{i}^{x}\left(t\right)=-G_{exc,i}^{x}\left(t\right)\left[V_{i}^{x}\left(t\right)-V_{exc}^{x}\right]-G_{inh,i}^{x}\left(t\right)\left[V_{i}^{x}\left(t\right)-V_{inh}^{x}\right] \end{array}$$ (34)

where *x* ∈ D1, D2 and $V_{exc}^{x}$, $V_{inh}^{x}$ denote the reversal potentials of excitatory and inhibitory synaptic currents, respectively.

Similarly, for a FSI neuron *i*, the excitatory conductance was:

$$\begin{array}{c} G_{exc,i}^{FSI}=\sum_{m\in K_{i}^{FSI}}\sum_{n}g_{exc}^{FSI}\left(t-t_{mn}^{Ctx}\right) \end{array}$$ (35)

and the total synaptic current for the FSI neuron *i* was

$$\begin{array}{c} I_{i}^{FSI}\left(t\right)=-G_{exc,i}^{FSI}\left(t\right)\left[V_{i}^{FSI}\left(t\right)-V_{exc}^{FSI}\right] \end{array}$$ (36)

The parameter values for both MSNs and FSIs in our network model are summarized in Table 3.

### Generation of B and W correlations

To separately control the correlations within and between the pre-synaptic pools of the striatal neurons, we extended the multiple-interaction process (MIP) model of correlated ensemble of Poisson type spike trains [12, 23]. The MIP model generates correlations by copying spikes from a spike train (the mother spike train) with a fixed probability (the copy probability, which determined the resulting correlation) to the individual spike trains. This process was implemented in the NEST simulator by introducing a synapse model that transmits spikes with a fixed probability: the lossy synapse. By making many convergent connections using the ‘lossy synapse’ we can mimic the random copying of spikes from the mother spike train to the children process. This way we controlled the correlations within (*W*) the pre-synaptic pool of a neuron.

**Fig 10:**
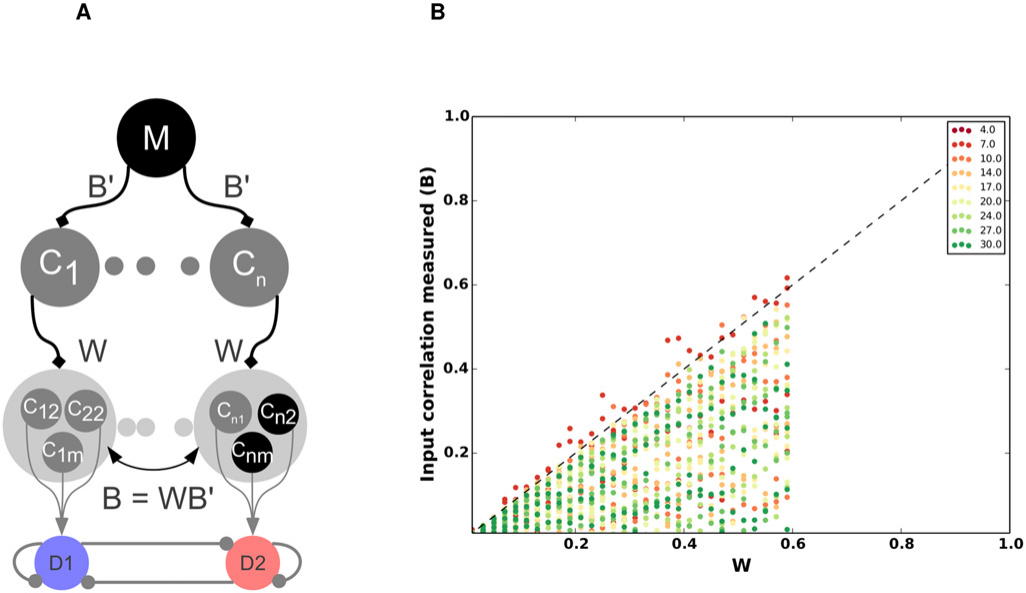
Generation of within and between correlations. **(A)** The correlations are generated by copying spikes twice from the mother process (M). Let *R*_*in*_ be the rate of the mother process. The spikes are copied into a set of children processes (*C*_1_ ⋯ *C*_*N*_) with a copy probability *B*′. This controls the shared input to the postsynaptic population and leads to a rate *R*_*in*_ *B*′. The spikes are copied for a second time with a copy probability *W* into a pool of input neurons (*C*_11_ ⋯ *C*_1*N*_). Each neuron in the input pool spikes with a rate *R*_*in*_ *B*′*W*. The correlation within the input pool can be increased by increasing *W*, whereas the shared input can be increased by increasing the copy probability *B*′. **(B)** *B* ≤ *W*. The means of the pairwise correlations measured from randomly chosen 2000 pairs of MSN neurons (*B*) are plotted against the corresponding *W* values. The different colors represent different input rates in Hz. Notice that most of the points lie below the diagonal.

To introduce correlations between (*B*) the pre-synaptic pools of two neurons we created a two-layered MIP (Fig 10). We started with a spike train *M* with a firing rate *R*_*in*_. In the first layer of MIP we generated *N* spike trains (*C*_1_, *C*_2_, ⋯, *C*_*N*_) with a fixed pair-wise correlation (*B*′) and firing rate (*R*_*in*_ *B*′). In the second layer, each of these spike trains acted as a mother spike train to generate pairwise correlations in the pre-synaptic pools of individual striatal neurons. Each spike train *C*_*i*_ was used to generate correlated spike trains (*C*_*i*0_, *C*_*i*1_, ⋯, *C*_*im*_) that acted as the pre-synaptic pool of each striatal neuron with a pairwise correlation *W* and firing rate *R*_*in*_ *B*′*W* (Fig 10). The effective correlation between the pre-synaptic pools of two neurons is then defined as *B* = *B*′*W*. Thus, in this model we can control the correlations between and within the pre-synaptic pools by changing the copy probabilities in the first and second layer.

### Relationship between *B* and *W*

*B* refers to the average correlation between the spiking activities of neurons in the pre-synaptic pools of two striatal neurons. The spike trains in each pre-synaptic pool are themselves correlated with a correlation coefficient *W*. For such pooled random variables, Bedenbaugh and Gerstein [22] derived the following relationship:

$$\begin{array}{c} \rho_{1,2}=\frac{B}{W+\frac{1}{n}\left(1-W\right)}+𝓞\frac{1}{n} \end{array}$$

where *ρ*_1,2_ is the correlation coefficient between the two pools of the pre-synaptic neurons, *n* is the size of each pre-synaptic pool. Because 0 ≤ *ρ*_1,2_ ≤ 1, it follows that *B* ≤ *W*.

Intuitively we can understand this relationship between *B* and *W* in the following way: Imagine two pools of identical spike trains, but with individual spikes trains uncorrelated to each other (i.e. *B*′ = 1 and *W* = 0). In this case, the average correlations between the two pools will be small because each spike train has only copy in the other pool, while being uncorrelated with all others. However, when *W* > 0, more such pairs with non-zero correlation will occur, increasing the value of *B*. Because, *B* ≤ *W*, the difference between the firing rates of the two MSN neuronal population in Fig 6 is estimated for the lower triangular region. This relationship is explicitly shown also in Fig 10B, where the measured input correlation between the pools (*B*) is shown to be less than *W*.

### Model limitations

Our model considers the striatum as a single action unit with ‘Go’ and ‘No-go’ counterparts and, hence, with a single DTT. But ideally, there are multiple channels of competing motor programs which can be envisioned as mutually competing DTT’s. Thus, future work will include the extension of this model to incorporate multiple action channels. Secondly, the effect of input correlations is yet not formulated into the analytical framework. This is a non-trivial problem, due to the non-linear transfer of input correlations into output firing rates, specifically if the output neurons are recurrently interconnected. Thirdly, our model explores the striatal dynamics at steady states, since the model showed stable fixed points. However, it is possible that in the presence of additional phenomena, such as plasticity of cortico-striatal or striato-striatal projections or intrinsic plasticity of the striatal neurons etc., the model may show interesting transient dynamics. Recent work proposed the existence of a ‘decision threshold’ in a different context [50]. They considered, the ‘decision threshold’ as a function of the RT (reaction time) distribution of the rats in a decision task pertaining to the transient dynamics of the basal ganglia. A future extension to this work can explore the effect of DTT due to the asymmetry in the striatal connectivity on the distance to the ‘decision threshold’ in [50], i.e. the effect of DTT on the RTs of a decision. And lastly, though our model abides by many experimental observations, it contradicts one experimental result, to the best of our knowledge. In [51], it was observed that FSIs increased their activity around a choice execution. In our model, a choice execution will correspond to the activation of the D1 pathway, which requires reduced activity in the FSIs, in contrast to the recordings in [51]. We think this contradiction is a consequence of our model of the striatum considering only one channel in the ‘Go’ and ‘No-Go’ pathways.

## Supporting Information

S1 Fig Effect of temporal fluctuations on DTT in the striatum. **(A)** Cortical input rate applied to the striatal mean field model. The instantaneous input rate was choosen from a low pass filtered noise (time constant = 5 msec) and standard deviation of ±0.5 around the mean. The mean of the input rate was varied every 500 msec while the standard deviation remained fixed. **(B)** To mimic a noisy Δ_*CTX*_ we provided additional noisy inputs to the D1 and D2 MSNs (Scenario I). D1 MSNs received additional input with a mean of 1 Hz and standard deviation *σ* − Δ − *ctx*1. **(C)** Similarly, the D2 MSNs also received low pass filtered noisy input with 0 Hz mean and standard deviation *σ* − Δ − *ctx*2. Both *σ* − Δ − *ctx*1 and *σ* − Δ − *ctx*2 were considered free variables here.**(D)** The instantaneous firing rates of D1 and D2 MSNs in response to the noisy cortical input and Δ_*CTX*_. A DTT can be observed in spite of noisy fluctuations. **(E)** To quantify the effect of noisy Δ_*ctx*_, on the DTT we measured the areas around the cross-over of the D1 and D2 MSNs firing rates. This is the area under the curve (yellow portion) for the time interval [t* − Δ,t*] (pre-DTT) and [t*,t* + Δ] (post-DTT), where t* is the time when DTT occurs. Because the gain of the D1 and D2 MSNs is different in pre-DTT (where λ_*D*1_ > λ_*D*2_) and post-DTT regions (where λ_*D*1_ < λ_*D*2_) we separately measured the area in the pre-DTT and post-DTT regimes. We normalised the pre-DTT and post-DTT areas with the areas measured for zero noise case. Here, we show the normalised pre-DTT and post-DTT areas for different values of *σ* − Δ − *ctx*1 and *σ* − Δ − *ctx*2. As expected the increase in the input noise progressively decreases the pre-DTT area. Nevertheless, we can reliably measure DTT for large fluctuations compared to the mean.
(TIFF)

S2 Fig Effect of striatal network connectivity parameters on the existence of the DTT. **(A-F)** Striatal parameters, Δ-ctx1, Δ-ctx2, *J*_11_, *J*_22_, *J*_21_, *J*_12_ were varied by up to ±100% around their means as specified in the model. It is difficult to visualise this six dimensional space. We use two different visualisations. First, we show whether for ±100% variation in one of the parameters it is possible to find a DTT for any set of values of all other parameters (again within ±100% of their means). The “Solution” refers to when a DTT is observed for at least one parameter set, given a specific value of the parameter for which robustness is tested. The values of parameters for which no combination of other parameters yielded a DTT were marked as “No solution”. It can be observed that a DTT can be found for nearly all values of all parameters (**A-F**). Only for very weak values of *J*_11_ and *J*_22_ we could not find a combination of other parameters that would yield a DTT. **(G-J)** Next, we show the existence DTT for pairs of striatum network parameters. Each dot shows the existence of a DTT. Not only do these solutions follow the relational constraints as described in Taverna et al. (2010) and the model (e.g. *J*_12_ > *J*_21_), but also the centroids (marker with red asterisk) of these clusters lie very close to the values used in the model. This can be verified in the mean values shown in (A-F). This indicates that these values are indeed a robust combination of striatal parameter values.
(TIFF)

S3 Fig Presence of DTT in spiking neural network in which D1 and D2 MSNs have different F-I curves. **(A)** F-I curves of D1 and D2 MSNs. Passive neuron properties were tuned to match the F-I curves of the D1 and D2 MSNs to match with the experimental measurement of the F-I curves of these neurons (shown in Gertler et al. 2008). **(B)** Firing rate of the D1 and D2 MSNs as a function of cortical input rate. D1 MSNs received extra input according to the scenario I. D1 and D2 MSNs have different F-I curves shown in the panel **A**. These results show that a DTT exists in the striatum even when D1 and D2 MSNs have different F-I curves. The network connectivity is same as described in Table 5.
(TIFF)

S4 Fig Effect of *B*′ correlations on striatum’s DTT. The blue and red regions mark the inputs for which D2 and D1 MSNs have higher firing rates. White regions show the inputs for which there is no difference between the firing rate of the two MSN populations. From these figures it is clear that *B*′ results in the increasing the region of high conflict.
(TIFF)

S5 Fig Effect of Dopamine and GPe firing rates on the balance of D1 and D2 activity (mean field equations). **(A)** Fixed points for D1 and D2 MSNs plotted for different levels of dopamine. Darker shades of blue (red) correspond to D1 (D2) MSN activity for higher levels of dopamine. For lower than normal levels of dopamine, the DTT shifts to the left (from ≈ 11 Hz to ≈ 6 Hz). This decreases the regime with the bias towards D1 MSNs. For higher than normal levels of dopamine, the DTT shifts to right (from ≈ 11 to ≈ 19Hz). This in turn, increases the regime with a bias towards D1 MSNs. **(B)** and **(C)** same as Fig 7 in the main text but calculated for mean field equations.
(TIFF)

S6 Fig Arbitration by GPe. **(A)** GPe activity is represented as an inhibition on FSI firing rates. A regime with a D2 bias in the rate equations is shown(λ_*D*1_ < λ_*D*2_, Δ_*MSN*_ < 0). At t = 400 ms, a constant inhibition is given to FSIs. The FSI activity decreases **(D)** and the bias shifts in the favour of D1 (λ_*D*1_ > λ_*D*2_, Δ_*MSN*_ > 0). **(B,E)** Shows the similar arbitration in spiking neural network activity. Dashed line represent the 95% confidence interval. **(C,F)** Inhibition on FSIs is unable to resolve the bias in favour of D1 in dopamine depletion conditions.
(TIFF)
