# Supplementary figures and images for: Existence and Control of Go/No-Go Decision Transition Threshold in the Striatum
Source: PLoS Comput Biol. 2015 Apr 24;11(4):e1004233. doi: 10.1371/journal.pcbi.1004233 (PMC4409064; doi:10.1371/journal.pcbi.1004233)

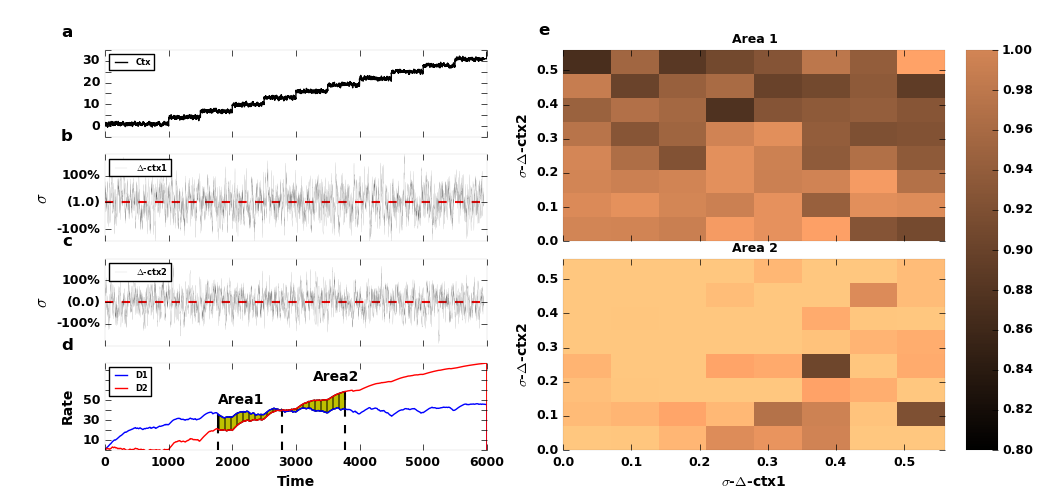

Supplement: S1 Fig — (A) Cortical input rate applied to the striatal mean field model. The instantaneous input rate was choosen from a low pass filtered noise (time constant = 5 msec) and standard deviation of ±0.5 around the mean. The mean of the input rate was varied every 500 msec while the standard deviation remained fixed. (B) To mimic a noisy ΔCTX we provided additional noisy inputs to the D1 and D2 MSNs (Scenario I). D1 MSNs received additional input with a mean of 1 Hz and standard deviation σ − Δ − ctx1. (C) Similarly, the D2 MSNs also received low pass filtered noisy input with 0 Hz mean and standard deviation σ − Δ − ctx2. Both σ − Δ − ctx1 and σ − Δ − ctx2 were considered free variables here.(D) The instantaneous firing rates of D1 and D2 MSNs in response to the noisy cortical input and ΔCTX. A DTT can be observed in spite of noisy fluctuations. (E) To quantify the effect of noisy Δctx, on the DTT we measured the areas around the cross-over of the D1 and D2 MSNs firing rates. This is the area under the curve (yellow portion) for the time interval [t* − Δ,t*] (pre-DTT) and [t*,t* + Δ] (post-DTT), where t* is the time when DTT occurs. Because the gain of the D1 and D2 MSNs is different in pre-DTT (where λD1 > λD2) and post-DTT regions (where λD1 < λD2) we separately measured the area in the pre-DTT and post-DTT regimes. We normalised the pre-DTT and post-DTT areas with the areas measured for zero noise case. Here, we show the normalised pre-DTT and post-DTT areas for different values of σ − Δ − ctx1 and σ − Δ − ctx2. As expected the increase in the input noise progressively decreases the pre-DTT area. Nevertheless, we can reliably measure DTT for large fluctuations compared to the mean. (TIFF) [file pcbi.1004233.s001.tiff]

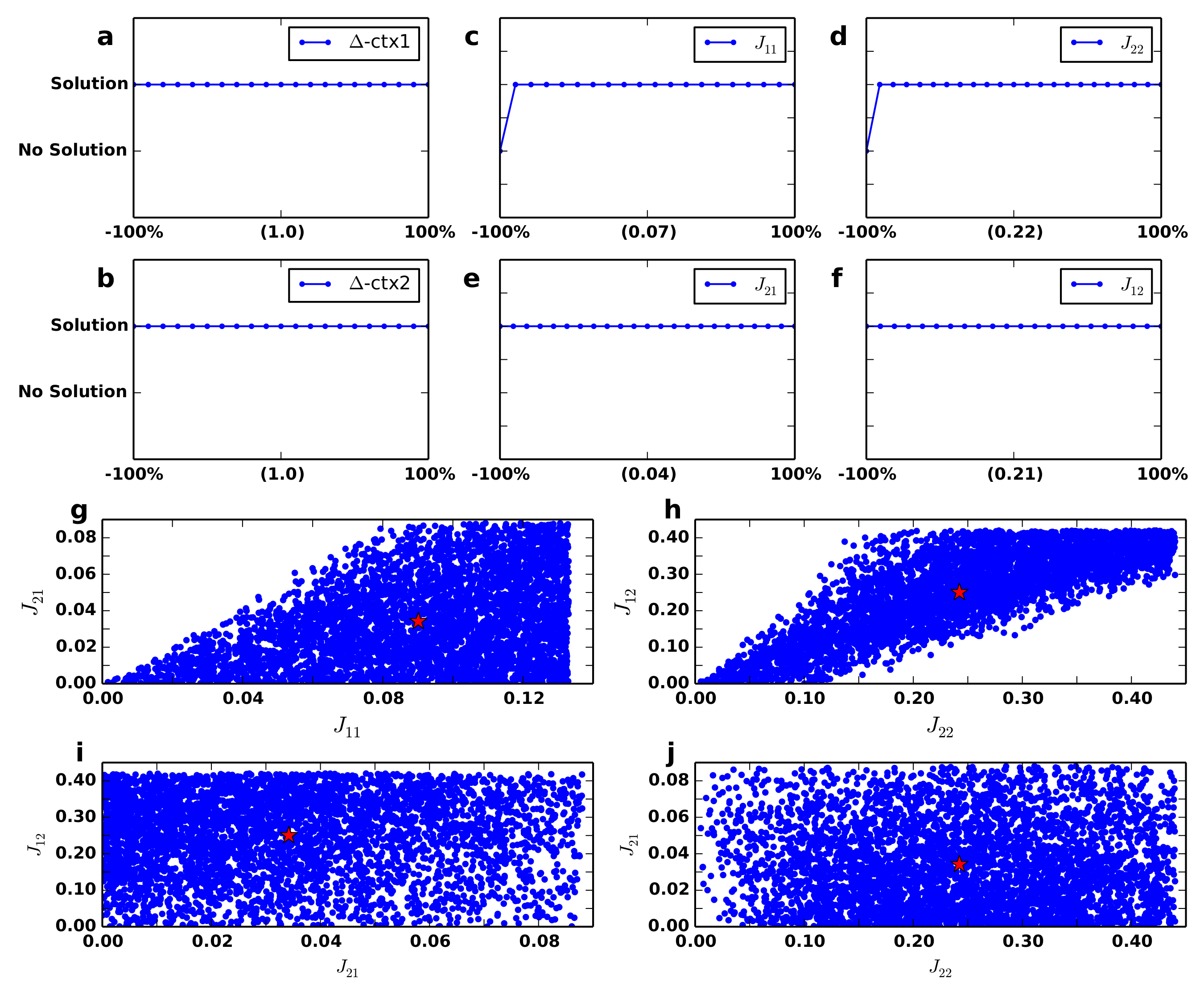

Supplement: S2 Fig — (A-F) Striatal parameters, Δ-ctx1, Δ-ctx2, J 11, J 22, J 21, J 12 were varied by up to ±100% around their means as specified in the model. It is difficult to visualise this six dimensional space. We use two different visualisations. First, we show whether for ±100% variation in one of the parameters it is possible to find a DTT for any set of values of all other parameters (again within ±100% of their means). The “Solution” refers to when a DTT is observed for at least one parameter set, given a specific value of the parameter for which robustness is tested. The values of parameters for which no combination of other parameters yielded a DTT were marked as “No solution”. It can be observed that a DTT can be found for nearly all values of all parameters (A-F). Only for very weak values of J 11 and J 22 we could not find a combination of other parameters that would yield a DTT. (G-J) Next, we show the existence DTT for pairs of striatum network parameters. Each dot shows the existence of a DTT. Not only do these solutions follow the relational constraints as described in Taverna et al. (2010) and the model (e.g. J 12 > J 21), but also the centroids (marker with red asterisk) of these clusters lie very close to the values used in the model. This can be verified in the mean values shown in (A-F). This indicates that these values are indeed a robust combination of striatal parameter values. (TIFF) [file pcbi.1004233.s002.tiff]

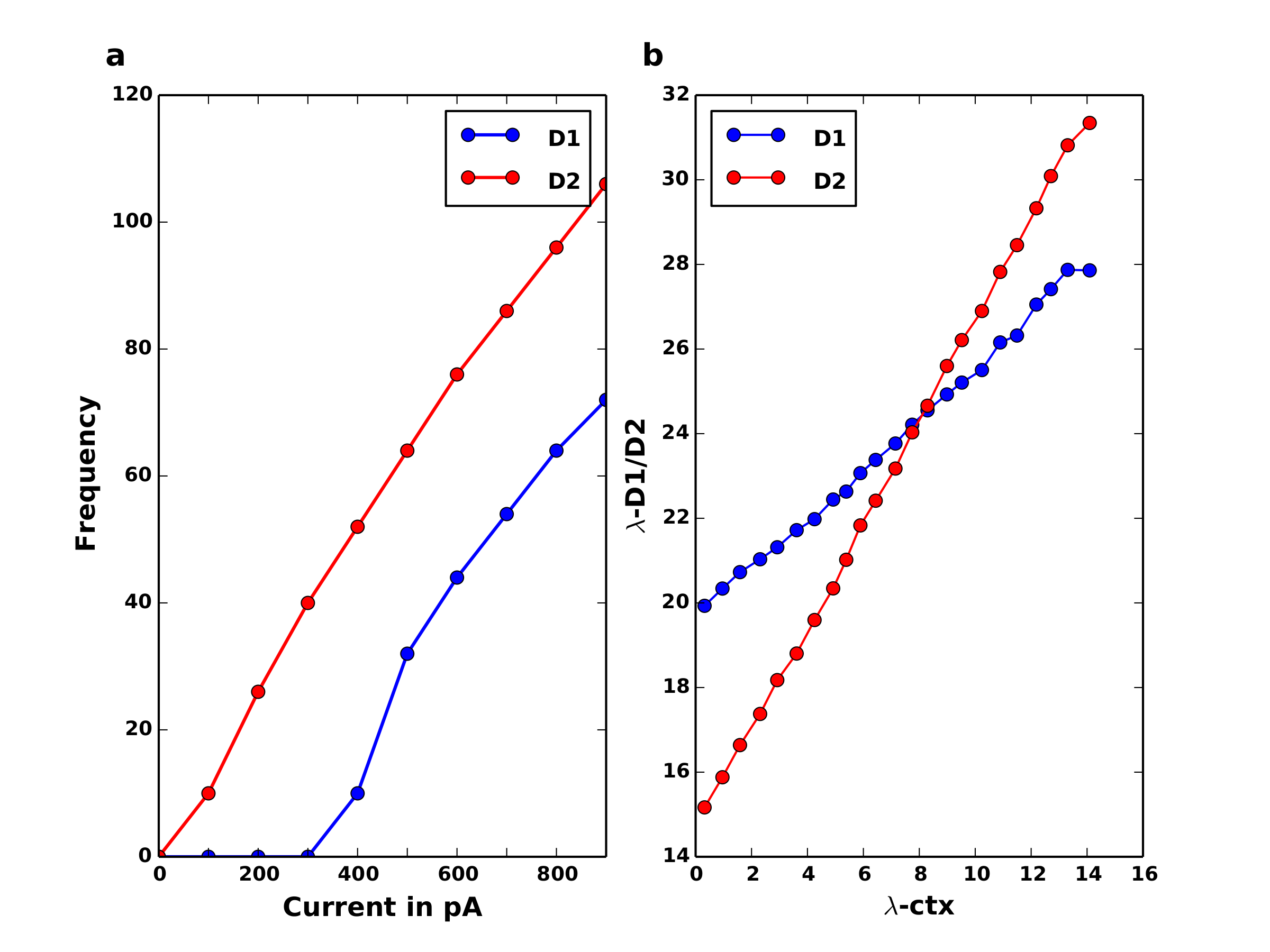

Supplement: S3 Fig — (A) F-I curves of D1 and D2 MSNs. Passive neuron properties were tuned to match the F-I curves of the D1 and D2 MSNs to match with the experimental measurement of the F-I curves of these neurons (shown in Gertler et al. 2008). (B) Firing rate of the D1 and D2 MSNs as a function of cortical input rate. D1 MSNs received extra input according to the scenario I. D1 and D2 MSNs have different F-I curves shown in the panel A. These results show that a DTT exists in the striatum even when D1 and D2 MSNs have different F-I curves. The network connectivity is same as described in Table 5. (TIFF) [file pcbi.1004233.s003.tiff]

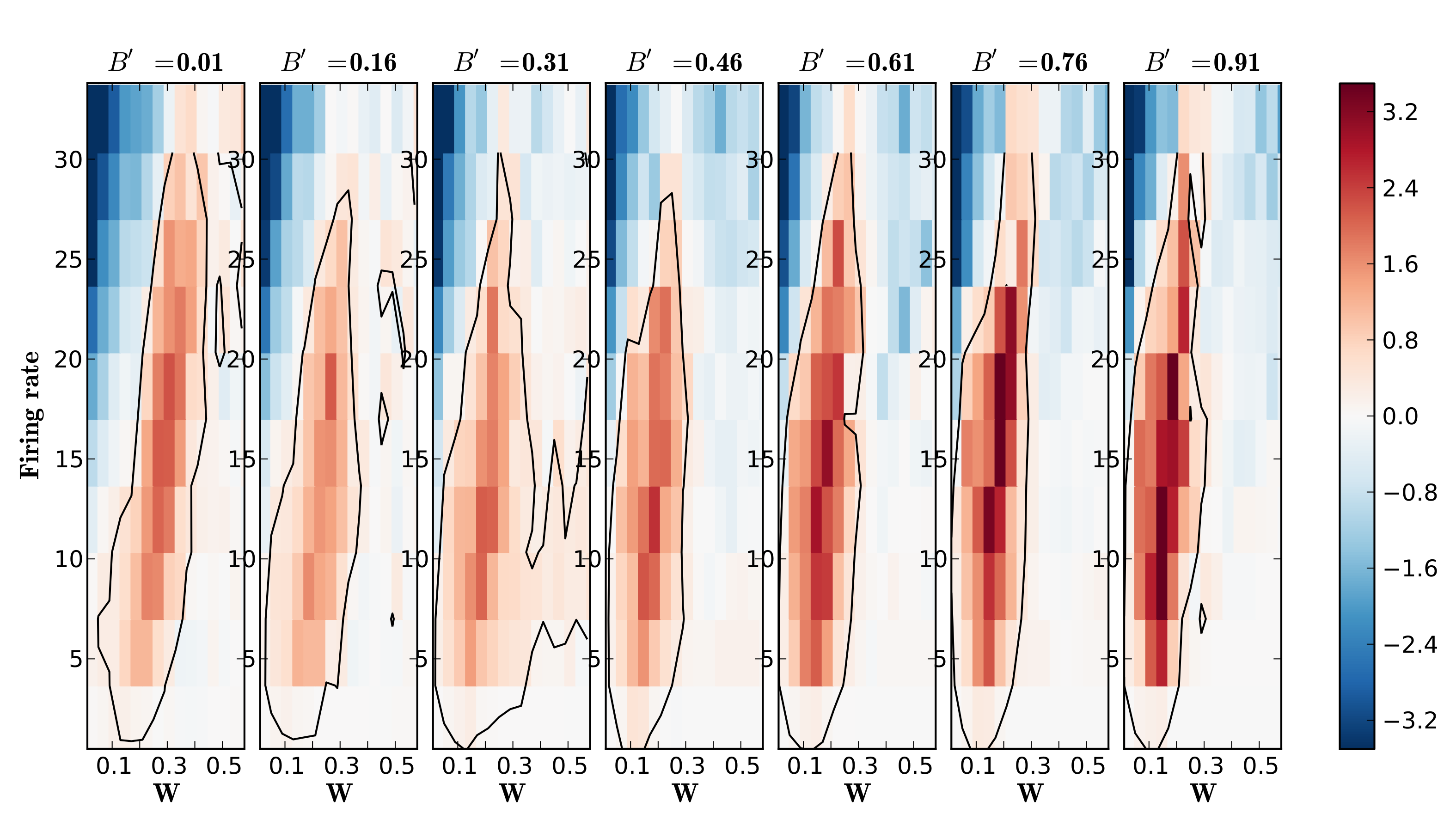

Supplement: S4 Fig — The blue and red regions mark the inputs for which D2 and D1 MSNs have higher firing rates. White regions show the inputs for which there is no difference between the firing rate of the two MSN populations. From these figures it is clear that B′ results in the increasing the region of high conflict. (TIFF) [file pcbi.1004233.s004.tiff]

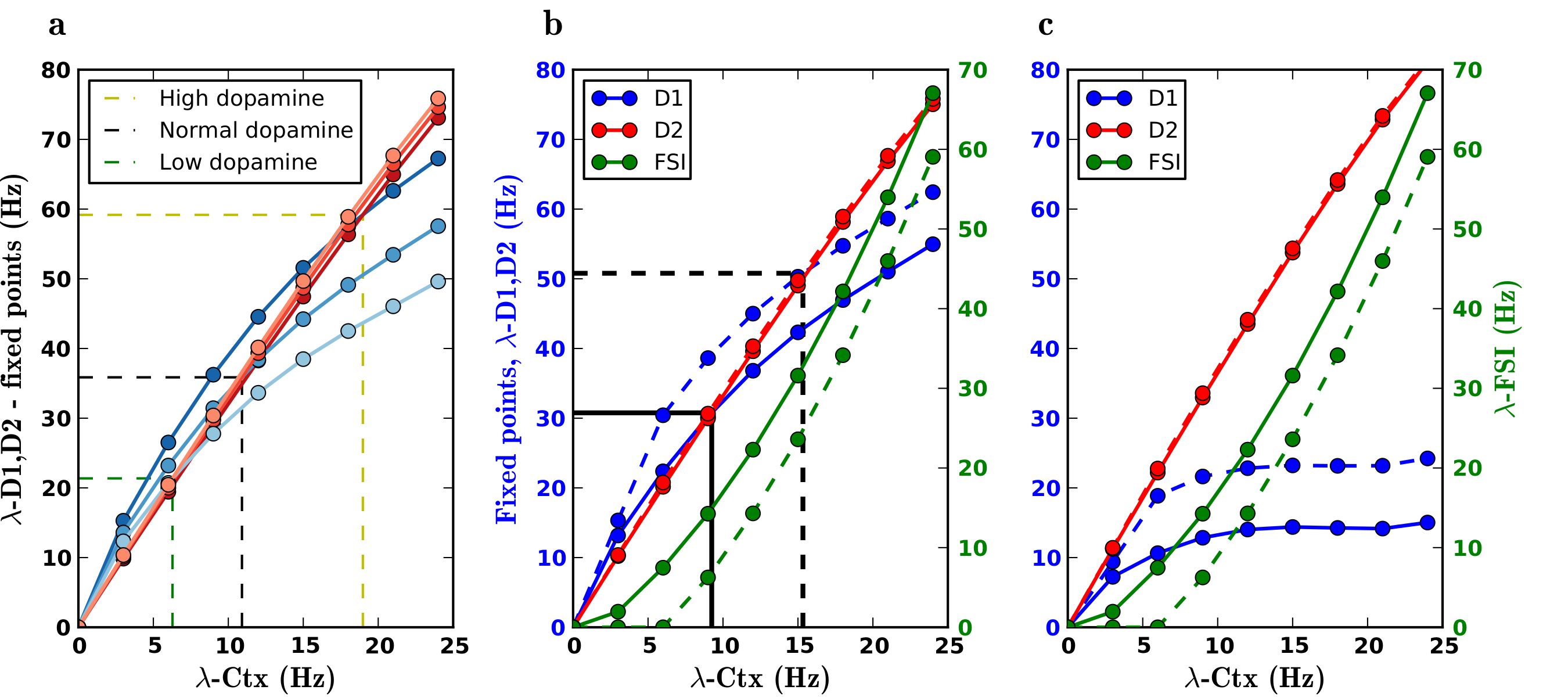

Supplement: S5 Fig — (A) Fixed points for D1 and D2 MSNs plotted for different levels of dopamine. Darker shades of blue (red) correspond to D1 (D2) MSN activity for higher levels of dopamine. For lower than normal levels of dopamine, the DTT shifts to the left (from ≈ 11 Hz to ≈ 6 Hz). This decreases the regime with the bias towards D1 MSNs. For higher than normal levels of dopamine, the DTT shifts to right (from ≈ 11 to ≈ 19Hz). This in turn, increases the regime with a bias towards D1 MSNs. (B) and (C) same as Fig 7 in the main text but calculated for mean field equations. (TIFF) [file pcbi.1004233.s005.tiff]

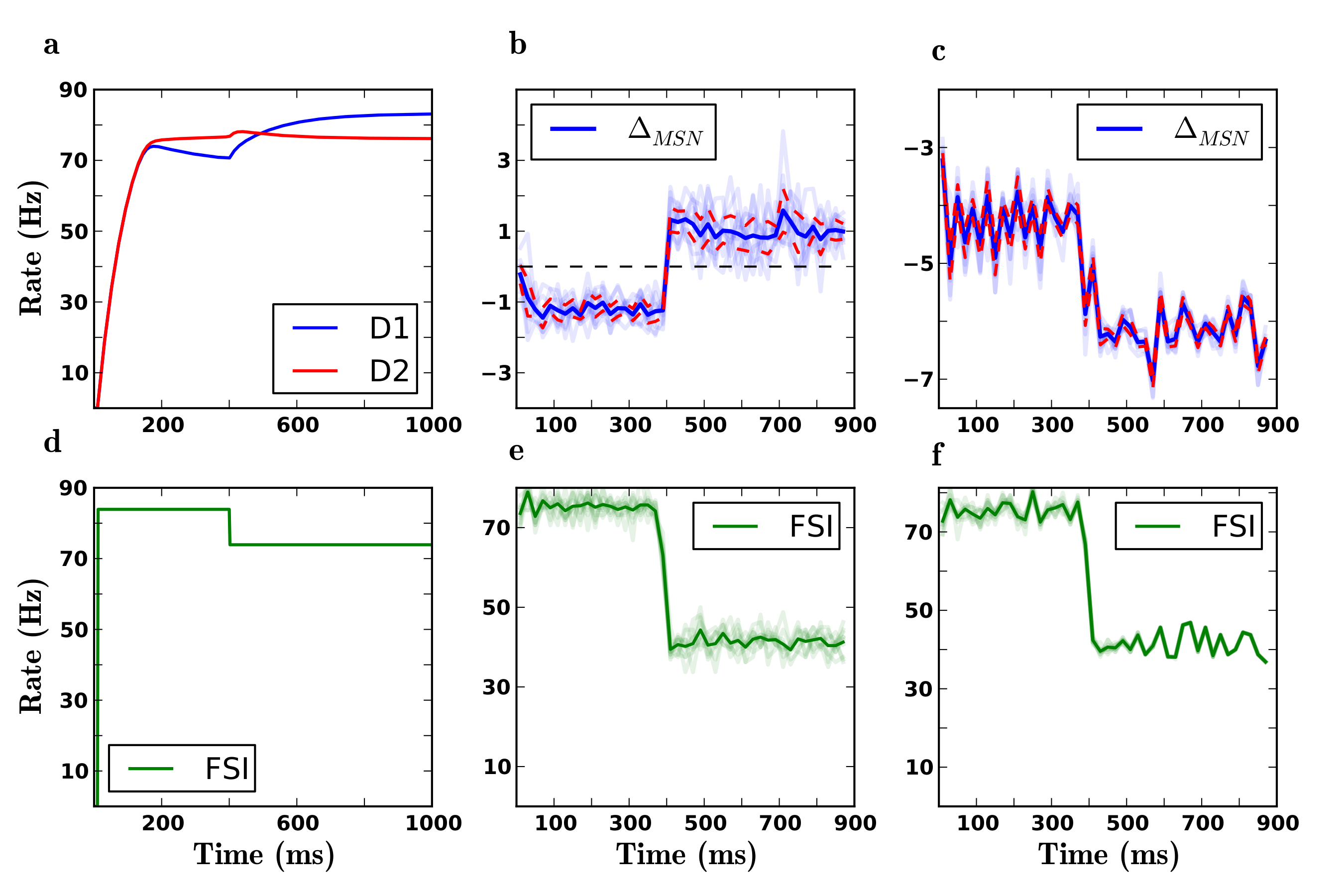

Supplement: S6 Fig — (A) GPe activity is represented as an inhibition on FSI firing rates. A regime with a D2 bias in the rate equations is shown(λD1 < λD2, ΔMSN < 0). At t = 400 ms, a constant inhibition is given to FSIs. The FSI activity decreases (D) and the bias shifts in the favour of D1 (λD1 > λD2, ΔMSN > 0). (B,E) Shows the similar arbitration in spiking neural network activity. Dashed line represent the 95% confidence interval. (C,F) Inhibition on FSIs is unable to resolve the bias in favour of D1 in dopamine depletion conditions. (TIFF) [file pcbi.1004233.s006.tiff]
